# Pan-Cancer Analysis of Alternative Lengthening of Telomere Activity

**DOI:** 10.3390/cancers12082207

**Published:** 2020-08-07

**Authors:** Ji-Yong Sung, Hee-Woong Lim, Je-Gun Joung, Woong-Yang Park

**Affiliations:** 1 Samsung Genome Institute, Samsung Medical Center, Seoul 06351, Korea; 5rangepineapple@gmail.com; 2 Department of Health Science and Technology, Samsung Advanced Institute of Health Science and Technology, Sungkyunkwan University, Seoul 06351, Korea; 3 Division of Biomedical Informatics, Cincinnati Children’s Hospital Medical Center, Cincinnati, OH 45229, USA; HeeWoong.Lim@cchmc.org; 4 Department of Molecular Cell Biology, School of Medicine, Sungkyunkwan University, Seoul 06351, Korea

**Keywords:** telomere maintenance mechanism, alternative lengthening of telomeres, cancers, gene expression, survival analysis

## Abstract

Alternative lengthening of telomeres (ALT) is a telomerase-independent mechanism that extends telomeres in cancer cells. It influences tumorigenesis and patient survival. Despite the clinical significance of ALT in tumors, the manner in which ALT is activated and influences prognostic outcomes in distinct cancer types is unclear. In this work, we profiled distinct telomere maintenance mechanisms (TMMs) using 8953 transcriptomes of 31 different cancer types from The Cancer Genome Atlas (TCGA). Our results demonstrated that approximately 29% of cancer types display high ALT activity with low telomerase activity in the telomere-lengthening group. Among the distinct ALT mechanisms, homologous recombination was frequently observed in sarcoma, adrenocortical carcinoma, and kidney chromophobe. Five cancer types showed a significant difference in survival in the presence of high ALT activity. Sarcoma patients with elevated ALT had unfavorable risks (*p* < 0.038) coupled with a high expression of *TOP2A*, suggesting this as a potential drug target. On the contrary, glioblastoma patients had favorable risks (*p* < 0.02), and showed low levels of antigen-presenting cells. Together, our analyses highlight cancer type-dependent TMM activities and ALT-associated genes as potential therapeutic targets.

## 1. Introduction

Telomeres are repetitive nucleoprotein structures located at the ends of chromosomes [1]. They play an essential role in protecting chromosome ends, preventing the DNA damage response (DDR) and maintaining genomic stability [2]. Telomere maintenance mechanisms (TMMs) differ between cancer cells and normal cells. As somatic cells divide, their telomeres are shortened, which ultimately activates cellular senescence and apoptosis. Unlike normal human somatic cells that have a finite proliferation capacity [3], cancer cells have an unlimited capacity to proliferate because of their distinct TMMs [4]. There are two TMM categories in human cancer: telomerase-mediated maintenance, which is observed in 80% of cancers, and alternative lengthening of telomeres (ALT) [5], which is found in ~15% of cancers [6,7]. ALT is based on the homologous recombination (HR)-dependent replication exchange and the synthesis of telomeric templates [8,9].

The unique characteristics of ALT include very long telomeres [10], telomere length heterogeneity [11], abundant extrachromosomal linear and circular telomere DNA [12], increased telomere-sister chromatid exchange (T-SCE) events [13], and the formation of ALT-associated promyelocytic leukemia (PML) bodies [14]. In addition, ALT-positive tumors have recurrent mutations in the alpha thalassemia/mental retardation syndrome X-linked (ATRX) gene [15] and the gene encoding the death domain associated (DAXX) protein [16]. Despite numerous studies on ALT, its clinical implications remain elusive. First, tumors with an ALT phenotype are associated with a poorer patient prognosis than ALT-negative tumors [17]. Second, these tumors are difficult to handle as they are recalcitrant tumors and have unlimited proliferation potential. Third, only a few anticancer drugs are available for the treatment of ALT tumors. Fourth, higher levels of mitochondrial dysfunction and reactive oxygen species (ROS) have been observed in ALT tumors, suggesting the potential of ALT as a therapeutic target [18].

Therefore, it is crucial to understand the molecular mechanisms underlying ALT and its impact on the survival of patients. To this end, we performed a thorough assessment of the connection between TMM-associated pathways and clinical prognostic indicators in various cancer types. We comprehensively analyzed TMM activities across 31 cancer types in The Cancer Genome Atlas (TCGA). Primarily, we focused on the common or distinct molecular features mediating the ALT pathways and assessed their clinical relevance. Furthermore, we investigated putative drug targets for ALT-active cancers. Understanding these TMM-associated markers and pathways may provide insights into ALT-related telomeric anomalies and the involvement of novel drug targets.

## 2. Results

### 2.1. TMM Activities Across 31 Cancer Types

**Figure 1:**
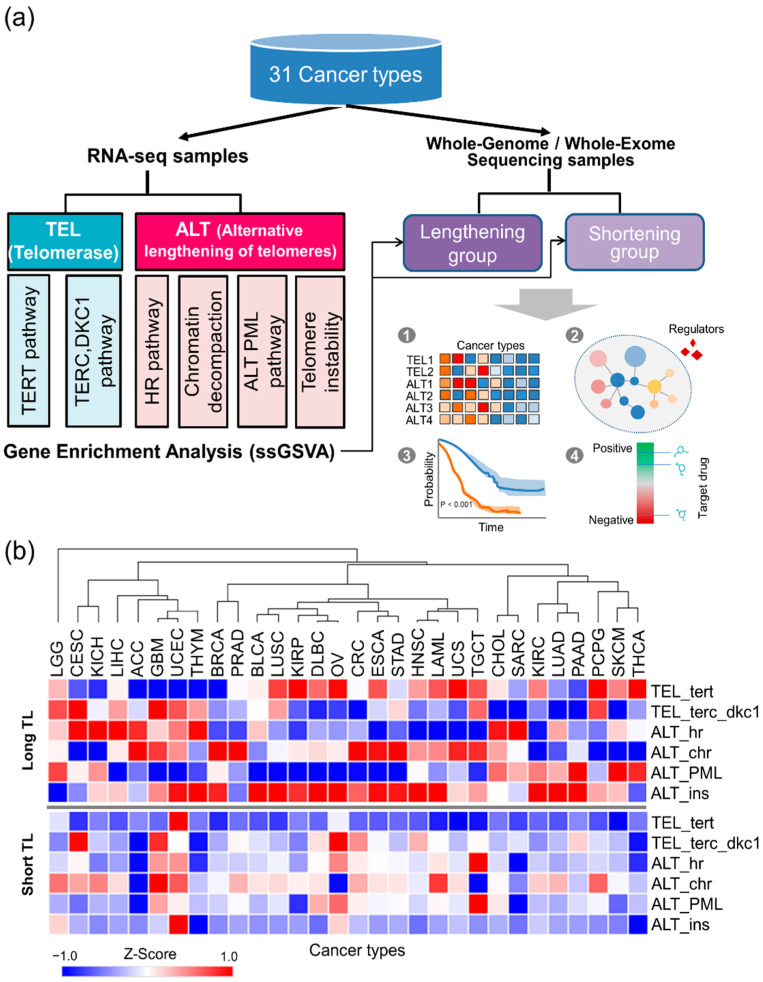
Telomere maintenance mechanism (TMM) analysis across 31 different cancer types. (**a**) Overview of our integrated TMM analysis pipeline using transcriptome profiles and telomere length from The Cancer Genome Atlas (TCGA) data. For RNA-seq data from 31 cancer types of TCGA, the activities of telomerase-associated pathways and alternative lengthening of telomeres (ALT)-associated pathways were inferred by utilizing single-sample gene set variation enrichment analysis (ssGSVA). Then, two groups of samples, i.e., telomere-lengthening (Long TL) samples and telomere-shortening (Short TL) samples, were examined for downstream analysis such as the analyses of enrichment patterns, transcriptional factors, survival analysis, and drug targets. (**b**) Enrichment of TMM pathways in Long TL samples, showing telomere elongation and Short TL samples, indicating telomere shortening. Tel_TERT, telomere TERT pathway; Tel_TERC_DKC1, telomere TERC DKC1 pathway; ALT_HR, ALT homologous recombination pathway; ALT_CHR, ALT chromatin decompaction pathway; ALT_PML, ALT-positive effect pathway; ALT_ins, ALT telomere instability pathway. A high Z-score indicates high activity in the corresponding TMM pathway.

To investigate the TMM activity in various cancer types, we analyzed RNA-seq data from 31 cancer types from the TCGA and examined information regarding their telomere length (Figure 1a, Table S1). Specifically, we considered two types of TMM mechanisms based on previously curated pathways [19]. First, ALT includes HR [5], chromatin decompaction, telomere instability, and the PML-related ALT pathways (Figure S1a and Table S2). Second, telomerase-associated pathways include the TERC pathway and the DKC1 and TERT pathways [19] (Figure S1b and Table S2). We utilized single-sample gene set variation enrichment analysis (ssGSVA) [20] to quantify the distinct TMM activities in each tumor. Then, we split the samples into two groups: telomere-lengthening (Long TL) samples and telomere-shortening (Short TL) samples, for each cancer type (Figure 1b).

Among a total of 8953 samples, 30% displayed Long TL compared to normal samples (Figure S2). Specifically, more than 50% of the sarcoma (SARC) samples were associated with telomere lengthening. Although telomere lengthening is mainly mediated by TERT (telomerase) and ALT (ATRX/DAXX alteration), approximately 22% of the samples might be affected by other mechanisms [21]. The telomerase and ALT activity in the Long TL group varied across cancer types. Overall, highly active TMM patterns were observed in all Long TL samples (Figure 1b). As expected, we found higher TMM activity in the Long TL group than in the Short TL group. Interestingly, several cancer types, including kidney chromophobe (KICH), adrenocortical carcinoma (ACC), breast invasive carcinoma (BRCA), prostate adenocarcinoma (PRAD), bladder urothelial carcinoma (BLCA), colon adenocarcinoma (CRC), SARC, lung adenocarcinoma (LUAD), and pancreatic adenocarcinoma (PAAD), displayed high ALT activity (*p* < 0.05) with low or no telomerase activity (Figure 1b). Three of these cancer types showed significantly greater enrichment of the ALT HR pathway in the Long TL group than in the Short TL group (SARC: *p* = 5.1 × 10^−10^, ACC: *p* = 4.4 × 10^−4^, KICH: *p* = 1.7 × 10^−4^). Overall, our analyses showed that both TMM pathways might be active in an individual sample, as previously suggested [22,23,24,25,26]. For example, both telomerase and ALT may be activated when the telomeres are considerably shortened [27].

### 2.2. Differential Gene Expression of the TMMs Reveals Different Cancer Hallmarks

**Figure 2:**
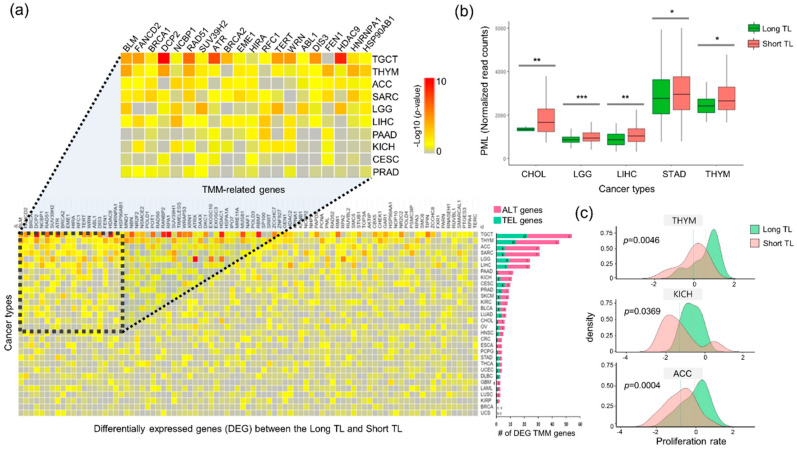
Differential gene expression of TMM reveals cancer types. (**a**) TMM-related genes differentially expressed in the Long TL samples and Short TL samples. Heat map showing the statistical significance of expression differences between the two groups, calculated using the Wilcoxon test and adjusted using the Benjamini–Hochberg procedure (*p* < 0.05, yellow; *p* ≤ 0.01, orange; *p* ≤ 0.001, red; *p* ≤ 0.0001, dark red). The top 30 samples were used for each group. The bar chart depicts the number of differentially expressed TMM genes (ALT-associated and TEL-associated) identified per cancer type. (**b**) Box plot representing the differences in *PML* expression between the Long TL and Short TL samples as a biomarker of TMM (* *p* < 0.05; ** *p* < 0.01; *** *p* < 0.001, Student’s *t*-test) (**c**) Density plot of the proliferation rate between the Long TL and Short TL samples. (ACC: *p* = 0.0004, KICH: *p* = 0.0369, and THYM: *p* = 0.0046). The proliferation rate was determined by predicting with the expression of proliferation-related genes. ACC, adrenocortical carcinoma; KICH, kidney chromophobe; THYM, thymoma.

To identify TMM-related genes, we performed differential gene expression analysis between the Long TL samples (*n* = 2684) and Short TL samples (*n* = 6269) for 30 different cancers using the gene expression data from tumor samples and matched normal samples. Several TMM-related genes were found to be abundant in testicular germ cell tumors (TGCT), thymoma (THYM), ACC, SARC, and brain lower grade glioma (LGG), in contrast to other tumor types (Figure 2a, Table S3). Of note, *BLM* and *FANCD2* were differentially expressed between the Long TL and Short TL groups in 30% (9/30) of cancer types. The Fanconi anemia protein, FANCD2, limits BLM-dependent telomere instability to inhibit telomere replication and recombination in human cells via the ALT pathway [28].

To further assess the roles of TMM in specific biological processes such as the P53 pathway and ROS generation, we also evaluated the correlation between the MSigDB cancer hallmark gene sets [29] and TMM (Figure S3). This analysis included 21 functional cancer hallmarks that have been implicated in TMM and cancer progression [30]. HR was positively correlated with ROS and G2M, and the ALT pathway was highly correlated with DNA repair, the P53 pathway, and ROS generation in the Long TL group of BRCA. Overall, these results suggest that TMM is intrinsically coupled with cancer hallmark pathways.

*PML* expression is an important characteristic of telomere maintenance. PML functions in various biological pathways as a tumor suppressor and as an ALT-related gene. PML plays an essential role in cell cycle regulation, survival, and apoptosis, and its inactivation or downregulation is frequently observed in cancer cells [31]. PML depletion induces telomere damage, nuclear and chromosomal abnormalities, and senescence [32]. It is commonly found in tumors with telomere shortening and high proliferation, which suggests its critical role in telomere maintenance and cell viability [33]. In our analysis, higher *PML* expression in the Short TL group than in the Long TL group was observed in five cancer types (STAD, THYM, hepatocellular carcinoma (LIHC), LGG, and cholangiocarcinoma (CHOL)) (Figure 2b), suggesting that *PML* expression level is a marker for telomere length in these cancer types. Its expression was significantly different between the Long TL and Short TL samples; however, its expression within each group of samples (Long TL or Short TL) was not significantly different (Figure S4a).

TMM in telomerase or ALT is required for unlimited tumor cell proliferation [34,35]. We found that the cell proliferation rate differs noticeably based on the telomere length in three cancer types (Figure 2c). In the Long TL group, the ALT HR pathway is positively correlated with proliferation rate in cervical squamous cell carcinoma and endocervical adenocarcinoma (CESC), lymphoid neoplasm diffuse large B-cell lymphoma (DLBC), kidney renal clear cell carcinoma (KIRC), PRAD, and uterine carcinosarcoma (UCS) (Figure S5). This suggests that these types of cancers may rely on the ALT mechanism utilizing homologous recombination during cellular proliferation.

### 2.3. Distinct TMM Pathways are Correlated with Prognosis in A Cancer Type-Specific Manner

**Figure 3:**
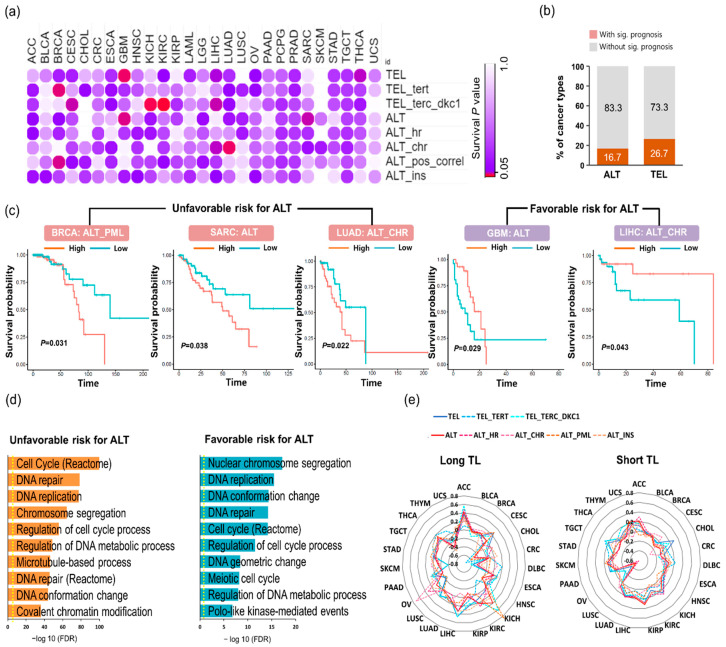
Molecular and clinical features of different TMMs affect prognosis in multiple cancer types. (**a**) Clinical associations of TMM with patient overall survival times. Color indicates statistical significance (*p* < 0.05, Red). (**b**) Percentages of cancer types with and without significant prognosis according to TEL and ALT activity, respectively. (**c**) Kaplan–Meier plots showing the overall survival rates for the high ALT group and low ALT group. The *p*-value was calculated using the log-rank test. Five cancer types (BRCA, SARC, LUAD, GBM, and LIHC) have significantly different prognoses. BRCA, breast invasive carcinoma; SARC, sarcoma; LUAD, lung adenocarcinoma; GBM, glioblastoma multiforme; LIHC, hepatocellular carcinoma (**d**) Gene ontology analysis of the differentially expressed genes (DEGs) between the high ALT group and low ALT group for unfavorable patient outcomes with high ALT in BRCA, SARC, and LUAD (left). Gene ontology analysis of high ALT and low ALT for favorable patient outcomes with high ALT in GBM and LIHC (right). (**e**) Spider map showing the correlation between TMM and tumor grade in the Long TL samples (left) and Short TL samples (right). The tumor grade information was obtained from available TCGA clinical data.

We examined the effects of the different types of TMMs on cancer prognosis. Historically, ALT-positive patients with sarcoma have been known to have poor prognoses [17], although patients with glioblastoma multiforme (GBM) have shown favorable outcomes [36,37]. The association between telomerase activity and patient survival has been studied previously [38,39]. In the present study, we comprehensively examined the relationships between distinct TMM pathways and clinical outcomes in 27 cancer types. We compared the survival rate of patients in the high TMM-score group and the low TMM-score group (Figure 3a). Nine cancer types with telomere lengthening, including BRCA, GBM, KICH, KIRC, LUAD, THCA, CESC, LIHC, and SARC, showed significant (*p* < 0.05) results for one or more types of TMM (Figure S6). Six (16.7%) and eight (26.7%) cancer types showed significant results according to ALT activity and TEL activity, respectively (Figure 3b). SARC and GBM with ALT displayed an opposite trend in survival rate (Figure 3c), consistent with previous studies [17,36,37]. LUAD showed a significant difference in ALT chromatin decompaction. Unexpectedly, high ALT chromatin decompaction (*p* = 0.043) was associated with a better prognosis for LIHC (Figure 3c). In addition, GBM had a good survival rate for high ALT (*p* = 0.029) as well as for high telomerase (*p* = 0.022). Many previous studies have demonstrated that patients with telomerase activity have a poorer prognosis. For example, a negative relationship was observed between telomerase activity and clinical outcome in breast cancer, colorectal cancer and gastrointestinal stromal tumors [38,40,41]. In gliomas, TERT promoter mutations lead to higher telomerase activity [42] but the associations between TERT promoter mutations and clinical outcomes are still controversial [43,44]. In the Short TL group, more cancer types also showed significantly different survival probabilities (Figure S7).

Next, we performed gene ontology analysis to obtain functional insights into ALT with regard to clinical outcomes. In the Long TL groups of BRCA, SARC, and LUAD, poor outcome-related biological pathways for high ALT were enriched with genes related to the cell cycle, DNA repair, and DNA replication (false discovery rate (FDR) < 0.001) (Figure 3d, Table S4). In GBM and LIHC, the poor outcome-related biological pathway was “*Staphylococcus aureus* infection,” (Figure S4b) and the good outcome-related biological pathway for high ALT was “nuclear chromosome segregation” (FDR < 0.001) (Figure 3d). The association of survival patterns with certain ALT pathways suggests the cancer type-specific functions of ALT.

We then checked the correlation between pathologic tumor stage and TMM pathway in two distinct TL groups (Figure 3e). We found a strong positive correlation between tumor stage and TMM scores in the Long TL groups of ACC and LIHC. In contrast, the tumor stages of CHOL and DLBC were negatively correlated with the ALT pathway. Furthermore, different correlation patterns were observed in the Short TL group, suggesting that TMM affects tumor development in different ways. For example, ovarian cancer was negatively correlated with all TMM scores but KICH and kidney renal papillary cell carcinoma (KIRP) were positively correlated with most TMMs. These results demonstrate that TMM may affect tumor progression differently according to TL and cancer type.

### 2.4. Vulnerabilities of ALT Activity for Cancer Therapy

**Figure 4:**
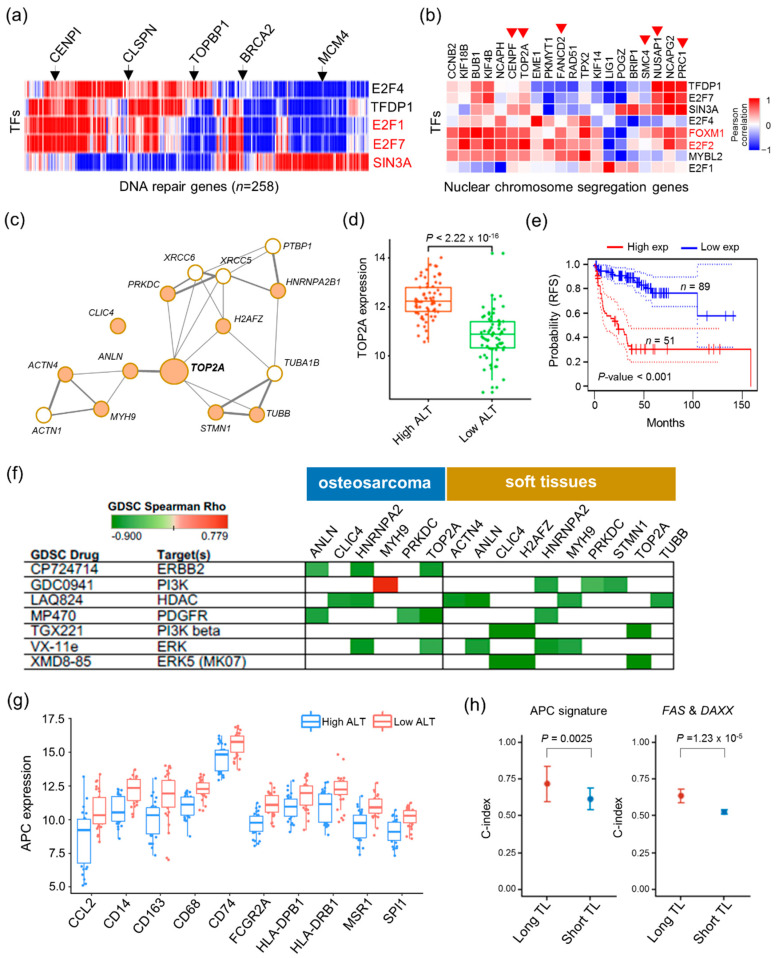
ALT activity correlates with therapeutic sensitivity. (**a**) Heat map of Pearson correlation coefficients between major transcription factors and target genes in the enriched biological pathway for three cancer types (BRCA, SARC, and LUAD) with high ALT. (**b**) Heat map of Spearman correlations between master transcription factors and target genes in the enriched biological pathways for two cancer types (GBM and LIHC) with high ALT. (**c**) Network for the top 10 differentially expressed and extended genes for sarcoma. (**d**) Box plot of *TOP2A* expression for distinct ALT activity in sarcoma. (**e**) Kaplan–Meier plot showing recurrence-free survival rates for high and low *TOP2A* expression in liposarcoma of the MSKCC cohort. (**f**) Genomics of Drug Sensitivity in Cancer (GDSC) drug and target genes for sarcoma molecular subtypes. Green (negative correlation) shows high drug sensitivity. (**g**) Comparison of the differentially expressed antigen-presenting cell (APC) signatures for high ALT and low ALT in GBM. (**h**) Power of risk prediction (C-index) with APC signature and *FAS* and *DAXX* genes for distinct telomere length in GBM.

We also examined the regulatory relationships between genes that belong to the prognosis-associated pathways in ALT. Cancers displaying unfavorable risk with high ALT (BRCA, SARC, and LUAD) may be regulated by the transcriptional factors *E2F4*, *TFDP1*, *E2F1*, *E2F7*, and *SIN3A* (FDR = 0.001) in the DNA repair pathway, including genes repairing DNA damage such as *CENPI*, *CLSPN*, *TOPBP1*, and *MCM4* (Figure 4a). They may also have potential regulatory roles in the cell cycle and DNA checkpoint signaling pathways in the telomere lengthening and ALT mechanisms. In contrast, cancers displaying favorable risk with high ALT (GBM and LIHC) may be regulated by *E2F4*, *TFDP1*, *FOXM1*, *E2F7*, *SIN3A*, *E2F1*, *MYBL2*, and *E2F2* (FDR = 0.0001) (Figure 4b). As hTERT repressors, *CTCF*, *E2F1*, and *SIN3A* showed significantly higher expression in high ALT tumors than in low ALT tumors (Figure S8). At high levels of ALT, these TFs may regulate the nuclear chromosome segregation pathway, in which many target genes are positively/negatively correlated with them.

Although there are many telomerase inhibitors, an anti-telomerase drug may convert the TMM from telomerase to ALT so that other cancer cells can escape death. ALT inhibitors that only target ataxia telangiectasia- and RAD3-related (ATR) kinase are currently available, although the effects of these ALT-targeting drugs are still controversial, especially with regard to their specificity [45,46]. There is an unmet need for the development of drugs for ALT tumors associated with telomere lengthening. We performed a drug-target prediction analysis using the Genomics of Drug Sensitivity in Cancer (GDSC) database [47]. The top-ranked significantly altered genes in high ALT were considered potential targets. Among these, *TOP2A* was more densely linked with other genes, indicating its functional importance as a hub gene (Figure 4c,d). We further examined its clinical relevance using another dataset, liposarcoma (MSKCC cohort: GSE30929), which revealed poor outcomes when it is highly expressed (Figure 4e). We identified candidate drugs (CP724714, MP470, TGX221, and XMD8-85) for *TOP2A* in molecular subtypes of sarcoma (osteosarcoma and soft tissue) (Figure 4f). We found that XMD8-85, as an ERK5 target, was also predicted in the Long TL samples in SARC by DeSigN (http://design.cancerresearch.my) (*p* = 0.032).

Tumor-associated macrophages with different immune signatures may also affect prognosis in glioblastoma. Indeed, patients with previously undefined TMM showed poor outcomes [48]. We showed that a strong immune response was enriched under conditions of low ALT levels for GBM and LIHC with poor patient outcomes (Figure S4c). Specifically, increased expression of antigen-presenting cell (APC) signature genes in the presence of low levels of ALT for glioblastoma could promote poor patient outcomes (Figure 4g). We also confirmed that the risk is higher (*p* = 0.0024) in the Long TL samples than in Short TL samples (Figure 4h), indicating that the TL lengthening group has greater clinical relevance.

In GBM, “post-operative *Staphylococcus aureus* infection” significantly affected patient survival [49]. We identified the protein association network of *Staphylococcus aureus* infection genes and ALT-related genes and found that *DAXX* and *FAS* were functionally connected (Figure S4b,d). *DAXX* is upregulated (Figure S4a), whereas *FAS* was downregulated at high levels of ALT. We compared the power of risk prediction by combining the expression of both genes between the Long TL group and Short TL group (Figure 4h). The prediction accuracy was significantly higher (*p* = 1.22 × 10^−5^) in the Long TL group than in the Short TL group, indicating a synergistic effect in the Long TL group.

## 3. Discussion

The unlimited replication of cells is one of the hallmarks of cancer [50]. In particular, ALT has been known to occur frequently in cancers of mesenchymal origin [6]. Although ALT occurs in sarcoma and some cancers, it is still unclear why ALT occurs only in certain cancer types. The high ALT activity in cancers of mesenchymal origin is also reflected in immortalized cell lines, many of which are fibroblasts. In a previous study, 22% of the TCGA data reported no TERT expression and no ATRX/DAXX mutation [21], which suggests an unknown TMM. Pan-cancer analysis for ALT suggests that ALT occurs in broad-spectrum tumors. Furthermore, the ALT mechanism not only depends on HR but can also be associated with other mechanisms such as telomere chromatin decompaction. The prognosis associated with TMM in different cancer types may also provide clues for harnessing the vulnerability of the TMM in the treatment of cancer patients.

As expected, ALT-related genes were highly expressed in the Long TL group of three cancer types (ACC, SARC, and KICH). We observed that telomeres had different TMMs according to the cancer type in each of the lengthening and shortening groups. Our results showed that telomere lengthening by the ALT mechanism is closely associated with the cell cycle and DNA checkpoint signaling pathways. TMMs are triggered to allow continued cell proliferation, and the ALT pathway in the DNA damage checkpoint pathway is associated with ATR-ATRIP, activating the *CHEK1* and *BRCA1* genes. Since activated RB1 would suppress E2F1, if RB1 fails to function, E2F1 is activated and enters the cell cycle pathway. E2F1 is also well known as a telomerase repressor (Figure S8) [51], but it can also play a role in the DDR pathway [52]. The family of E2F transcription factors includes the activator E2F1 and the repressor E2F4, which cooperate to facilitate a proper transition through the cell cycle [53,54].

Telomeres use both the telomerase and ALT mechanisms simultaneously to deal with the DNA replication crisis when the telomeres have become extremely short [27]. Since cancers evolve aggressively, they keep their telomeres long as they become malignant. Telomere shortening could lead to entry into the senescence and apoptosis pathways in the DDR, but ALT blocks both apoptosis and the cell senescence pathways via the p53 pathway. It was shown that a TMM such as HR was activated in three cancer types with no or low telomerase activity and may be regulated by the same master regulators.

The regulation of E2Fs and senescence by PML nuclear bodies is vital for ALT processes [55]. Numerous target genes of E2F are interconnected with the DNA repair and DNA damage checkpoints. In their absence, cells accumulate DNA damage signals that induce p53 activity and the senescence process [56]. In the Long TL group, HR genes were overexpressed relative to the Short TL group. We also compared *PML* expression by telomere length and found that *PML* expression was higher in the Short TL group. In some cancer types, high *PML* expression is associated with telomere shortening for tumor suppression and apoptosis, cell cycle arrest, and senescence, owing to the lack of telomere maintenance [31].

The ALT mechanism also appears to be associated with the prognostic outcomes of cancers. Interestingly, GBM and LIHC had better survival probabilities with higher ALT activities, whereas SARC, LUAD, and BRCA had better survival probabilities with lower ALT activities. Prognosis depends on the ALT activity of the tissue type in telomere elongation. Functional analysis of the genes associated with higher ALT suggests that the nuclear chromosome segregation that increases aneuploidy plays a beneficial role in GBM and LIHC, unlike in other cancer types. In GBM, signatures of antigen-presenting cells increased in the presence of low levels of ALT, and are associated with poor patient outcomes. Novel therapeutic strategies that consider the tumor microenvironment may be potentially useful for glioblastoma patients with low levels of ALT. However, despite the good correlation between ALT activities and survival rates in these cancer types, there is a limitation due to the number of samples showing ALT. Further assessment with large cohorts may be required to arrive at concrete conclusions.

We investigated the potential targets for cancers with ALT activity by utilizing a drug sensitivity database. In sarcoma, TOP2A and ERK5 were inferred as potential targets. Since only a few ALT-specific drugs such as ALT inhibitors targeting ATR kinase are currently known, it is noteworthy that our proposed novel targets may contribute to personalized therapies according to the TMM. Future research is urgently needed to evaluate drugs for cancers associated with ALT, and as a first step, candidate target drugs should be validated by in vivo and in vitro assays such as analyses using cell lines or patient-derived xenograft (PDX) models.

## 4. Materials and Methods

### 4.1. Data Sets

We obtained mRNA normalized expression data (version 2016.8.16; Platform: IlluminaHiSeq_RNASeqV2) for 31 cancer types including five pooled sets (BRCA, SARC, LUAD, GBM, and LIHC) from Broad GDAC Firehose (https://gdac.broadinstitute.org/). We utilized the telomere lengths (TLs) calculated using TelSeq [56] software from whole genome sequencing (WGS) data and whole exome sequencing (WES) data from a previous study [21]. The relative TL ratio was defined as tumor telomere length/normal telomere length (tTL/nTL), following a previous study [21], i.e., the tTL divided by the nTL, corresponding to the pair-matched TL ratio information. If the relative TL ratio of a tumor was greater than 1, it was assigned to the “Long TL” group. If the ratio was less than 1, it was assigned to the “Short TL” group. The workflow for the data analysis is illustrated in Figure 1a. In addition, clinical information, including tumor grade information [57] and data measuring the proliferation rate [58] were used for downstream analyses. The proliferation rate was predicted by the expression of genes significantly associated with it.

### 4.2. TMM Signature Analysis

We focused on six main TMM pathways and signature gene sets [19]. The activities of the pathways for each sample were obtained by single-sample gene set enrichment (ssGSEA) in the GSVA R package [20], using RNA-seq expression profiles. In the pre-processing step of mRNA expression, genes with RSEM (RNA-Seq by expectation-maximization) expression levels of <1 in >50% of samples were removed, and these levels were log_2_-transformed. To assess the significance of the scores, we estimated *p* values by generating the background distribution from perturbed expression profiles (1,000,000 times).

### 4.3. Differential Expression Gene Analysis of Cancer Types

We performed DEG analysis for the Long TL samples compared to the Short TL samples, as well as the samples with high ALT compared to the samples with low ALT in 31 cancer types, using the Limma R package [59]. Scale normalization and moderated Student′s *t*-tests were performed using empirical Bayes statistics in the Limma package [59]. The resulting *p* values were adjusted for multiple testing using the false discovery rate, Benjamini and Hochberg correction method.

### 4.4. Gene Ontology and Correlation Analysis

Gene ontology analysis was performed using METASCAPE [60], with DEG (FDR < 0.05). We used Pearson′s correlation coefficient (r) between TMM pathways and proliferation, tumor grade, and cancer hallmark in each cancer type. Gene–gene correlation analysis was performed using pairwise Pearson’s correlation coefficient. Correlation heat maps were visualized using Morpheus software (https://software.broadinstitute.org/morpheus/).

### 4.5. Transcription Factor Analysis Protein Association Network

We identified transcription factors (TFs) and target genes using the Cytoscape plug-in, iRegulon, which pairs motifs and chromatin immunoprecipitation-sequencing (ChIP-seq) tracks to determine the TFs controlling gene networks, and the iRegulon database (version 2015.02.12) [61]. Briefly, Cytoscape networks were created by importing the list of DEGs. The set of nodes (genes) was submitted to iRegulon and analyzed using the following options: (1) motif collection (10 kb region, 9713 position-weight matrices (PWMs)), (2) track collection (1120 ChIP-seq tracks of ENCODE uniform signals), (3) putative regulatory region (20 kb centered around TSS), (4) motif rankings database (20 kb region centered around TSS, 7 species), and (5) track of rankings database (20 kb region centered around TSS, ChIP-seq derived). We filtered TF targets with low correlation (enrichment score threshold 3.0, maximum FDR on motif similarity, FDR: 0.001). The functional protein association network was constructed through the input of multiple genes of significant DEGs using the STRING tool with high confidence and was extended by adding nodes connected to an initial network [62].

### 4.6. Survival Probability Analysis

The R package, ‘‘survival’’ [63] was used to perform the overall survival analysis and produce the Kaplan–Meier survival plots. A log-rank test was used to assess the significance (*p* < 0.05). The powers of risk prediction (C-index) were measured using the coxph function in the survival R package (survAUC). We performed prediction analysis using 20-fold cross-validation. We determined candidate drugs that could be sensitive in patients with high ALT in SARC using data from GDSC [47]. The Limma [59] output for DEG analysis was used to search for drug target genes. The signature (upregulated genes) was plugged into GDSC to identify drugs of interest.

## 5. Conclusions

We successfully demonstrated that TMMs work differently depending on the type of cancer. Furthermore, ALT activation could affect the prognosis of patients with specific cancer types. These results provide valuable insights for the development of precision medicine to treat aggressive tumors.

## Supplementary Materials

The following are available online at https://www.mdpi.com/2072-6694/12/8/2207/s1, Figure S1: Overall pathways of genes involved in the telomere maintenance mechanism (TMM). (a) ALT-associated pathways (four sub-pathways: HR, Chromatin Decompaction, PML, and Telomere Instability) (b) Telomerase-associated pathways (two sub-pathways: TERT and TERC_DKC1), Figure S2: Bar graph showing the frequency distribution of the Long TL samples (n = 2684) and Short TL samples (n = 6269) from TCGA. SARC is top-ranked as it had a large number of telomere lengthening samples, while UVM had a small number of telomere lengthening samples, Figure S3: Correlation between seven key cancer hallmarks (G2M, EMT, DNA REPAIR, P53, ROS, HYPOXIA, and ANGIOGENESIS) and telomere maintenance mechanisms. (a) In telomere lengthening group. (b) In telomere shortening group. The brown color shows positive correlation and the blue color indicates negative correlation, Figure S4: Different ALT-related phenotypes in cancer types (see Figure 2b) with unfavorable risk for ALT. (a) Significant TMM-associated genes between high and low levels of ALT. (b) Functional protein association network between Staphylococcus aureus infection and TFs in low ALT types (GBM and LIHC). (c) The bar plot of enriched biological process terms in low ALT cancer types (GBM and LIHC). (d) Heatmap of correlations between Staphylococcus aureus infection and ALT related genes, Figure S5: Heatmap of correlations between proliferation rates and TMMs in the telomere-lengthening and -shortening groups from 31 cancer types (Red color: FDR < 0.05). The Pearson’s correlation coefficient between proliferation rates and TMM scores was calculated across all samples for each cancer type, Figure S6: Kaplan–Meier plots of overall survival for the telomere-lengthening group in seven cancer types (KIRC, CESC, KICH, GBM, LIHC, BRCA, and THCA), Figure S7: Kaplan–Meier plots of overall survival for the telomere-shortening group in nine cancer types (ACC, LGG, BRCA, HNSC, KIRC, KIRP, LIHC, PAAD, and THYM). Significant survival differences were observed in ALT-associated pathways, Figure S8: Box plot comparing the expression of hTERT repressors (CTCF, E2F1, and SIN3A). (a) In the unfavorable risk group associated with ALT in three cancer types (BRCA, LUAD, and SARC) (b) In the favorable risk group associated with ALT in two cancer types (GBM and LIHC), Table S1. List of 31 TCGA cancer types, Table S2. List of genes involved in the telomere maintenance mechanism, Table S3. List of significant signature genes involved in the telomere maintenance mechanism across cancer types, Table S4. List of significant gene ontology (GO) terms enriched in the high ALT and low ALT groups.
