# Supplementary material for: Pan-Cancer Analysis of Alternative Lengthening of Telomere Activity
Source: Cancers (Basel). 2020 Aug 7;12(8):2207. doi: 10.3390/cancers12082207 (PMC7465155; doi:10.3390/cancers12082207)
Supplement: Supplementary file 1 [file cancers-12-02207-s001.pdf]

Supplementary Materials: Pan-Cancer Analysis of Alternative Lengthening of Telomere Activity

Ji-Yong Sung, Hee-Woong Lim, Je-Gun Joung and Woong-Yang Park

a

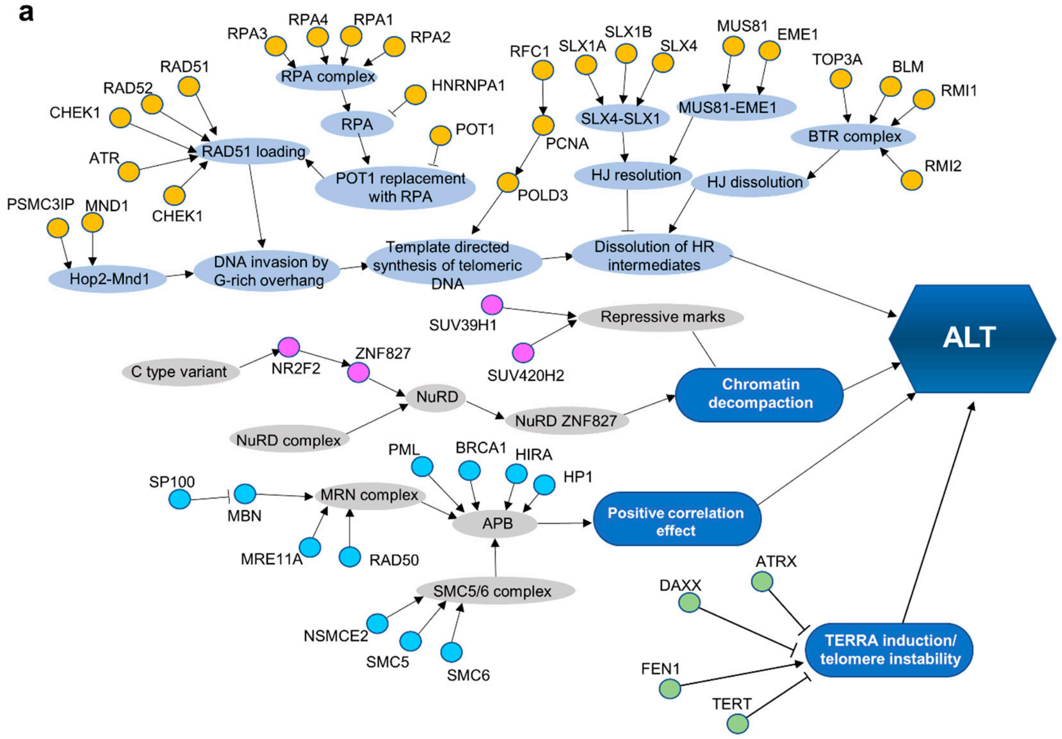

b

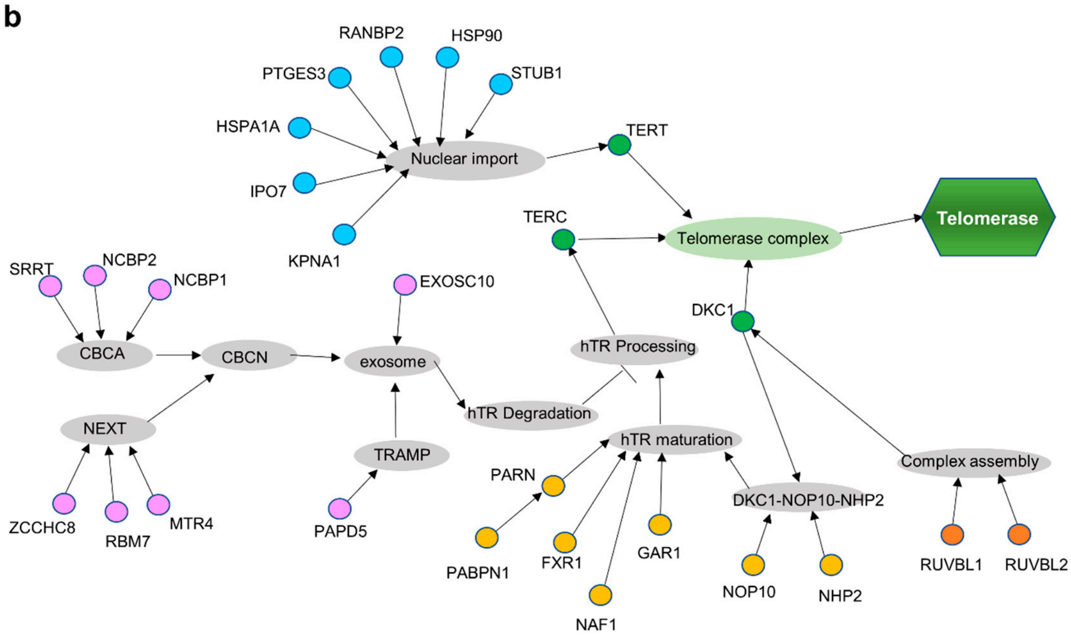

Figure S1. Overall pathways of genes involved in the telomere maintenance mechanism (TMM). **(a)** ALT−associated pathways (four sub−pathways: HR, Chromatin Decompaction, PML, and Telomere Instability) **(b)** Telomerase−associated pathways (two sub−pathways: TERT and TERC_DKC1).

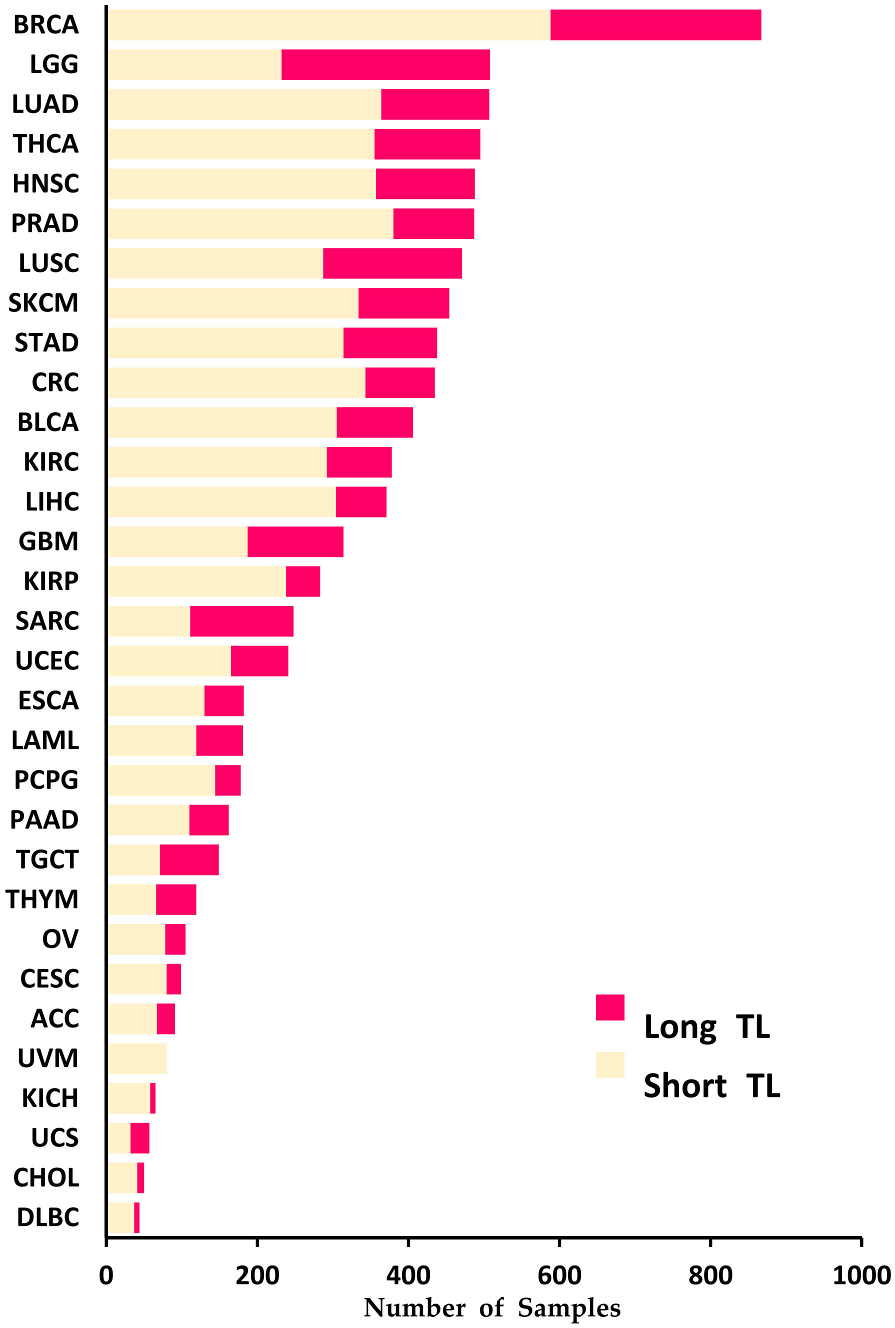

Figure S2. Bar graph showing the frequency distribution of the Long TL samples (*n* = 2,684) and Short TL samples (*n* = 6,269) from TCGA. SARC is top-ranked as it had a large number of telomere lengthening samples, while UVM had a small number of telomere lengthening samples.

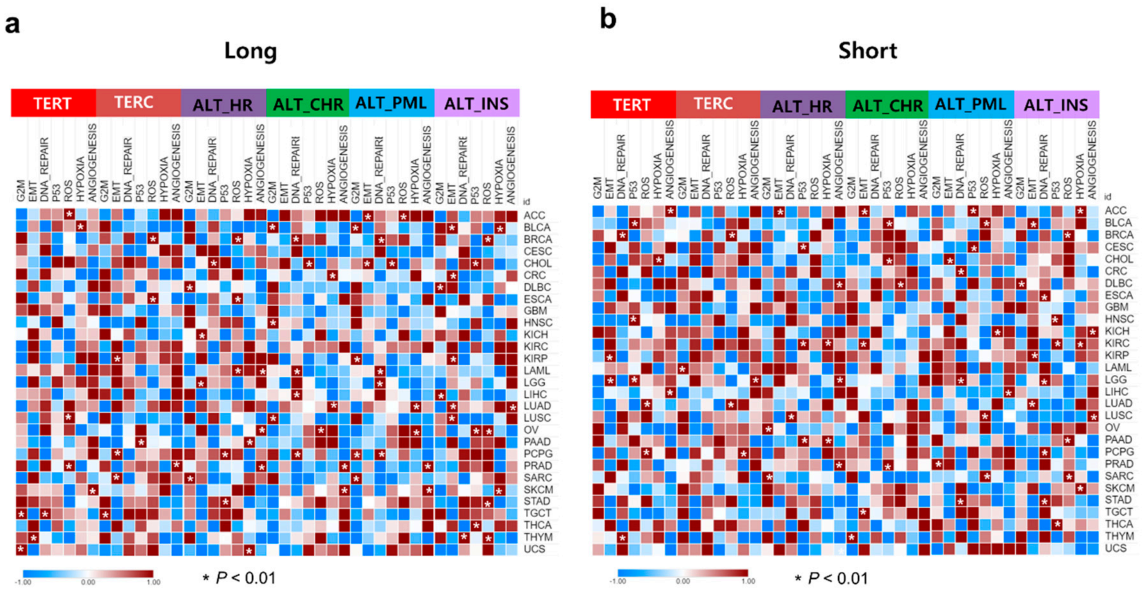

Figure S3. Correlation between seven key cancer hallmarks (G2M, EMT, DNA REPAIR, P53, ROS, HYPOXIA, and ANGIOGENESIS) and telomere maintenance mechanisms. (a) In telomere lengthening group. (b) In telomere shortening group. The brown color shows positive correlation and the blue color indicates negative correlation.

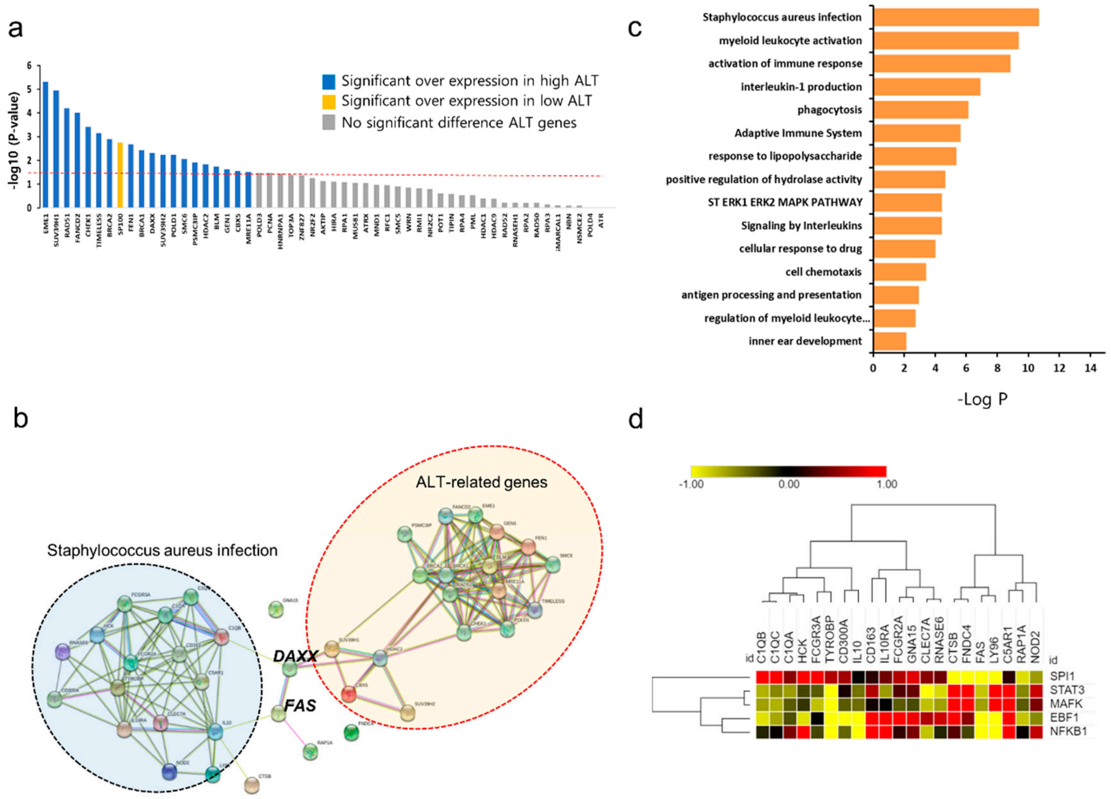

Figure S4. Different ALT−related phenotypes in cancer types (see Figure S2b) with unfavorable risk for ALT. **(a)** Significant TMM−associated genes between high and low levels of ALT. **(b)** Functional protein association network between *Staphylococcus aureus infection* and TFs in low ALT types (GBM and LIHC). **(c)** The bar plot of enriched biological process terms in low ALT cancer types (GBM and LIHC). **(d)** Heatmap of correlations between *Staphylococcus aureus infection* and ALT related genes.

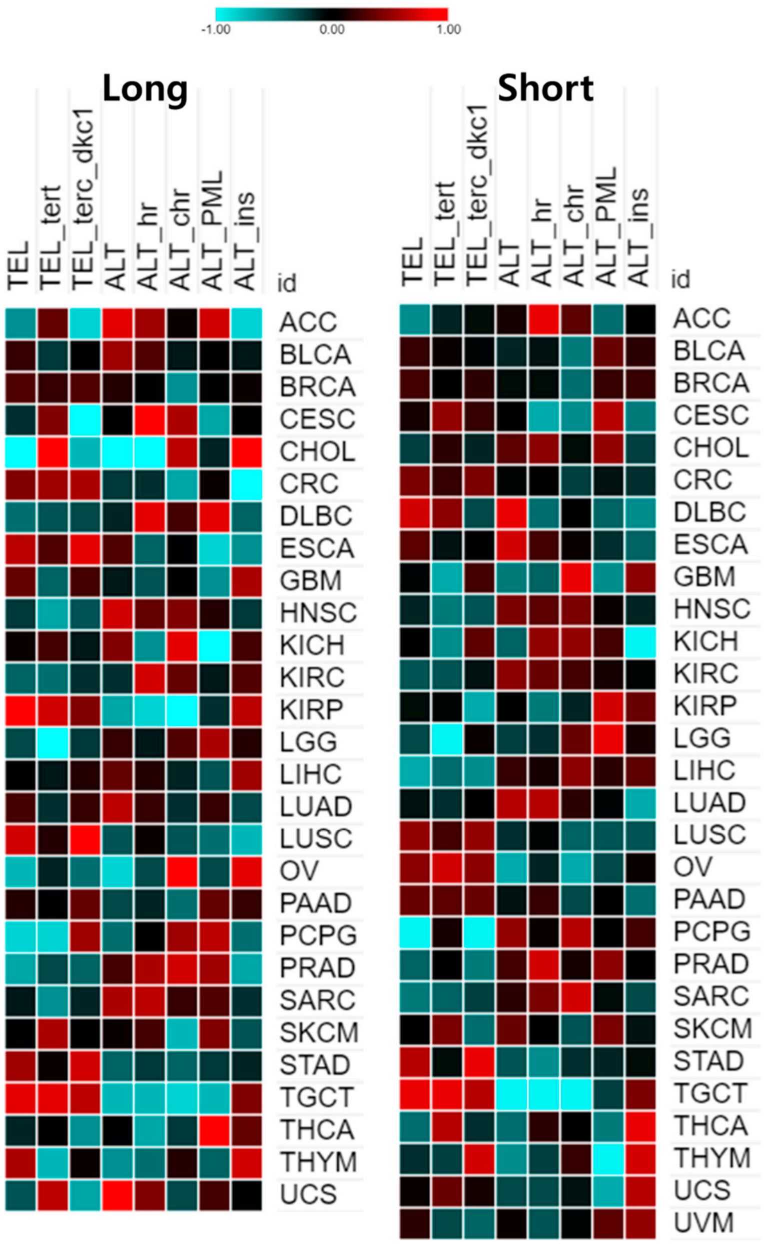

Figure S5. Heatmap of correlations between proliferation rates and TMMs in the telomere−lengthening and −shortening group from 31 cancer types (Red color: FDR< 0.05).

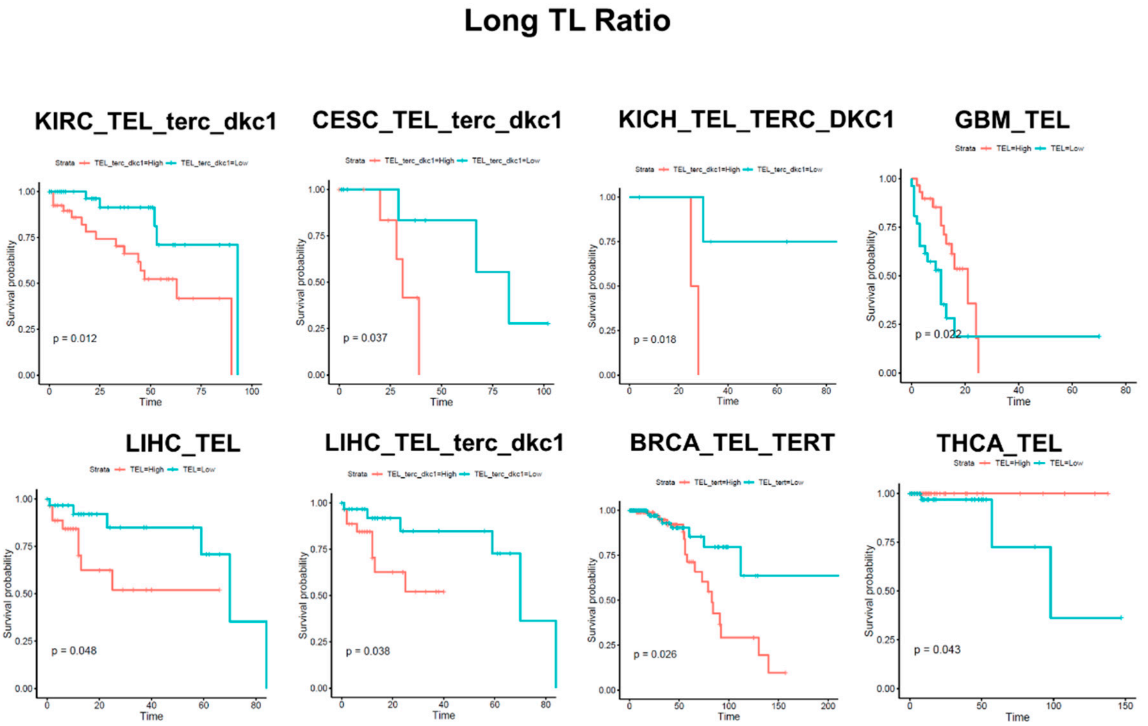

Figure S6. Kaplan−Meier plots of overall survival for the telomere lengthening group in seven cancer types (KIRC, CESC, KICH, GBM, LIHC, BRCA, and THCA).

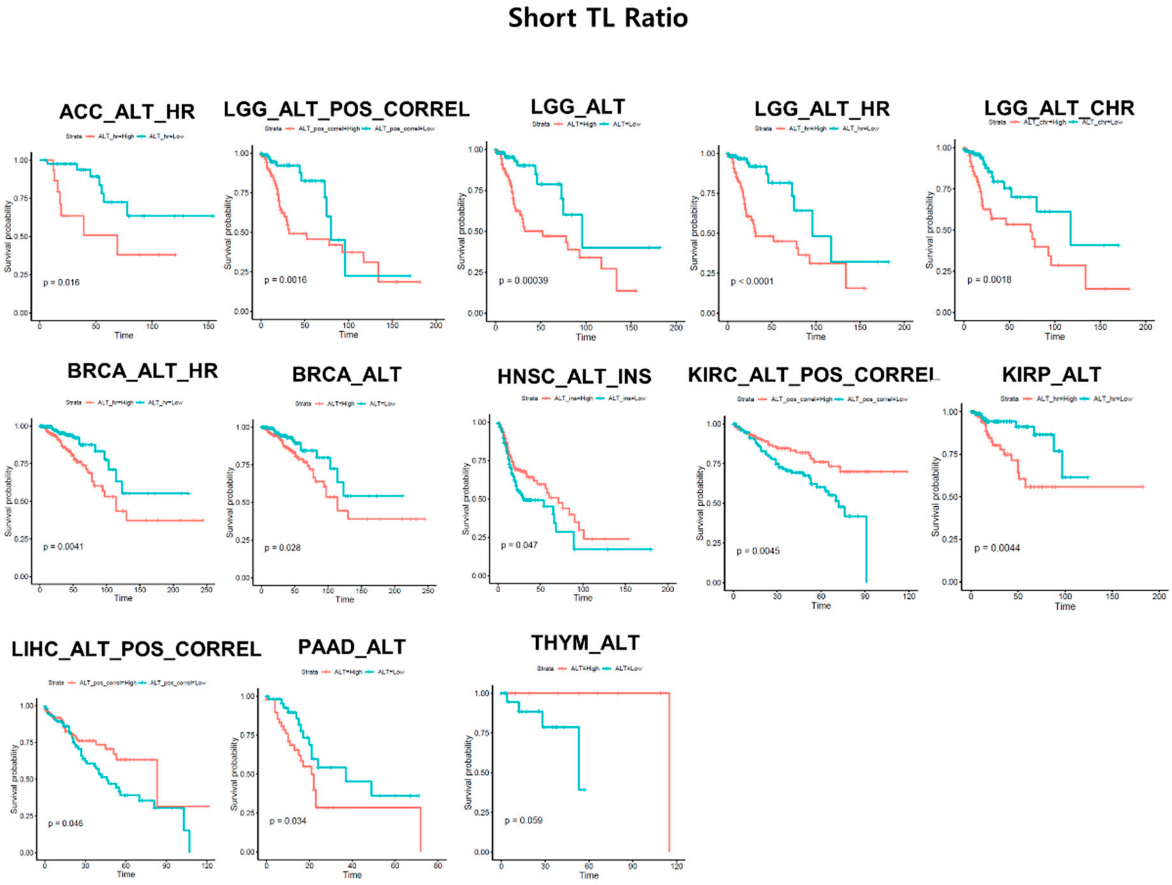

Figure S7. Kaplan−Meier plots of overall survival for the telomere shortening group in nine cancer types (ACC, LGG, BRCA, HNSC, KIRC, KIRP, LIHC, PAAD, and THYM). Significant survival differences were observed in ALT−associated pathways.

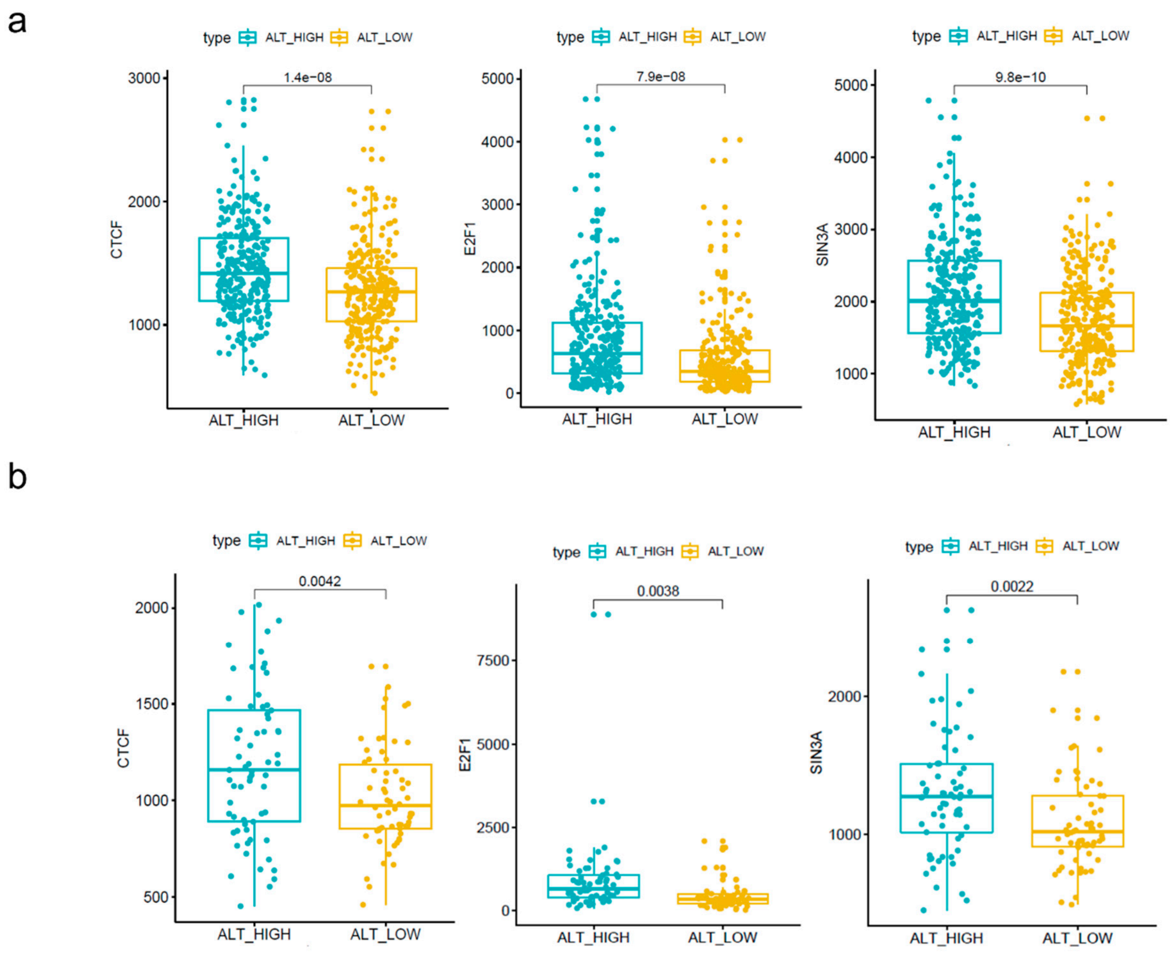

Figure S8. Box plot comparing the expression of hTERT repressors (CTCF, E2F1, and SIN3A). **(a)** In the unfavorable risk group associated with ALT in three cancer types (BRCA, LUAD, and SARC). **(b)** In the favorable risk group associated with ALT in two cancer types (GBM and LIHC).

Table S1. List of 31 TCGA cancer types.

Abbreviation	Full Name	Cases
ACC	Adrenocortical Carcinoma	91
BLCA	Bladder Urothelial Carcinoma	406
BRCA	Breast invasive carcinoma	867
CESC	Cervical squamous cell carcinoma and endocervical adenocarcinoma	99
CHOL	Cholangiocarcinoma	50
CRC	Colon adenocarcinoma	435
DLBC	Lymphoid Neoplasm Diffuse Large B−cell Lymphoma	44
ESCA	Esophageal carcinoma	182
GBM	Glioblastoma multiforme	314
HNSC	Head and Neck squamous cell carcinoma	488
KICH	Kidney Chromophobe	56
KIRC	Kidney renal clear cell carcinoma	378
KIRP	Kidney renal papillary cell carcinoma	283
LAML	Acute Myeloid Leukemia	181
LGG	Brain Lower Grade Glioma	508
LIHC	Liver hepatocellular carcinoma	371
LUAD	Lung adenocarcinoma	507
LUSC	Lung squamous cell carcinoma	471
OV	Ovarian serous cystadenocarcinoma	105
PAAD	Pancreatic adenocarcinoma	162
PCPG	Pheochromocytoma and Paraganglioma	178
PRAD	Prostate adenocarcinoma	487
SARC	Sarcoma	248

SKCM	Skin Cutaneous Melanoma	454
STAD	Stomach adenocarcinoma	438
TGCT	Testicular Germ Cell Tumors	149
THCA	Thyroid carcinoma	495
THYM	Thymoma	119
UCEC	Uterine Corpus Endometrial Carcinoma	241
UCS	Uterine Carcinosarcoma	57
UVM	Uveal Melanoma	80

Table S2. List of genes involved in the telomere maintenance mechanism.

TMM type	Pathway	Gene Name	Description
Alternative Lengthening Telomere	Homologous Recombination pathway	*WRN*	WRN RecQ like helicase
Alternative Lengthening Telomere	Homologous Recombination pathway	*AKTIP*	AKT Interacting Protein
Alternative Lengthening Telomere	Homologous Recombination pathway	*POLD4*	DNA Polymerase Delta 4, Accessory Subunit
Alternative Lengthening Telomere	Homologous Recombination pathway	*POLD3*	DNA Polymerase Delta 3, Accessory Subunit
Alternative Lengthening Telomere	Homologous Recombination pathway	*POLD1*	DNA Polymerase Delta 1, Catalytic Subunit
Alternative Lengthening Telomere	Homologous Recombination pathway	*CBX5*	Chromobox 5
Alternative Lengthening Telomere	Homologous Recombination pathway	*HIRA*	Histone Cell Cycle Regulator
Alternative Lengthening Telomere	Homologous Recombination pathway	*FANCD2*	FA Complementation Group D2
Alternative Lengthening Telomere	Homologous Recombination pathway	*TIMELESS*	Timeless Circadian Regulator
Alternative Lengthening Telomere	Homologous Recombination pathway	*TIPIN*	TIMELESS Interacting Protein
Alternative Lengthening Telomere	Homologous Recombination pathway	*SMARCAL1*	SWI/SNF Related, Matrix Associated, Actin Dependent Regulator Of Chromatin, Subfamily A Like 1
Alternative Lengthening Telomere	Homologous Recombination pathway	*PCNA*	Proliferating Cell Nuclear Antigen
Alternative Lengthening Telomere	Homologous Recombination pathway	*CHEK1*	Checkpoint Kinase 1
Alternative Lengthening Telomere	Homologous Recombination pathway	*RNASEH1*	Ribonuclease H1
Alternative Lengthening Telomere	Homologous Recombination pathway	*MND1*	Meiotic Nuclear Divisions 1
Alternative Lengthening Telomere	Homologous Recombination pathway	*RAD51*	RAD51 Recombinase
Alternative Lengthening Telomere	Homologous Recombination pathway	*ATR*	ATR Serine/Threonine Kinase

Alternative Lengthening Telomere	Homologous Recombination pathway	*RAD52*	RAD52 Homolog, DNA Repair Protein
Alternative Lengthening Telomere	Homologous Recombination pathway	*SLX1A*	SLX1 Homolog A, Structure−Specific Endonuclease Subunit
Alternative Lengthening Telomere	Homologous Recombination pathway	*SLX1B*	SLX1 Homolog B, Structure−Specific Endonuclease Subunit
Alternative Lengthening Telomere	Homologous Recombination pathway	*SLX4*	SLX4 Structure−Specific Endonuclease Subunit
Alternative Lengthening Telomere	Homologous Recombination pathway	*BLM*	BLM RecQ like helicase
Alternative Lengthening Telomere	Homologous Recombination pathway	*RMI1*	RecQ Mediated Genome Instability 1
Alternative Lengthening Telomere	Homologous Recombination pathway	*RMI2*	RecQ Mediated Genome Instability 2
Alternative Lengthening Telomere	Homologous Recombination pathway	*PSMC3IP*	PSMC3 Interacting Protein
Alternative Lengthening Telomere	Homologous Recombination pathway	*TOP3A*	DNA Topoisomerase III Alpha
Alternative Lengthening Telomere	Homologous Recombination pathway	*POT1*	protection of telomeres 1
Alternative Lengthening Telomere	Homologous Recombination pathway	*RPA4*	Replication Protein A4
Alternative Lengthening Telomere	Homologous Recombination pathway	*RPA3*	Replication Protein A3
Alternative Lengthening Telomere	Homologous Recombination pathway	*HNRNPA1*	Heterogeneous Nuclear Ribonucleoprotein A1
Alternative Lengthening Telomere	Homologous Recombination pathway	*RPA2*	Replication Protein A2
Alternative Lengthening Telomere	Homologous Recombination pathway	*FEN1*	Flap Structure−Specific Endonuclease 1
Alternative Lengthening Telomere	Homologous Recombination pathway	*RPA1*	Replication Protein A1
Alternative Lengthening Telomere	Homologous Recombination pathway	*RFC1*	Replication Factor C Subunit 1
Alternative Lengthening Telomere	Homologous Recombination pathway	*BRCA2*	BRCA2 DNA Repair Associated
Alternative Lengthening Telomere	Chromatin Decompaction pathway	*NR2C2*	Nuclear Receptor Subfamily 2 Group C Member 2
Alternative Lengthening Telomere	Chromatin Decompaction pathway	*NR2F2*	nuclear receptor subfamily 2 group F member 2
Alternative Lengthening Telomere	Chromatin Decompaction pathway	*SUV39H2*	Suppressor Of Variegation 3−9 Homolog 2

Alternative Lengthening Telomere	Chromatin Decompaction pathway	*SUV39H1*	Suppressor Of Variegation 3−9 Homolog 1
Alternative Lengthening Telomere	Chromatin Decompaction pathway	*HDAC1*	histone deacetylase 1
Alternative Lengthening Telomere	Chromatin Decompaction pathway	*HDAC2*	histone deacetylase 2
Alternative Lengthening Telomere	Chromatin Decompaction pathway	*ZNF827*	Zinc Finger Protein 827
Alternative Lengthening Telomere	Chromatin Decompaction pathway	*KMT5C*	Lysine Methyltransferase 5C
Alternative Lengthening Telomere	Chromatin Decompaction pathway	*KMT5B*	Lysine Methyltransferase 5B
Alternative Lengthening Telomere	Chromatin Decompaction pathway	*HDAC9*	Histone Deacetylase 9
Alternative Lengthening Telomere	PML pathway	*SP100*	SP100 Nuclear Antigen
Alternative Lengthening Telomere	PML pathway	*BRCA1*	BRCA1 DNA Repair Associated
Alternative Lengthening Telomere	PML pathway	*PML*	Promyelocytic Leukemia
Alternative Lengthening Telomere	PML pathway	*GEN1*	GEN1 Holliday Junction 5' Flap Endonuclease
Alternative Lengthening Telomere	PML pathway	*MUS81*	MUS81 Structure−Specific Endonuclease Subunit
Alternative Lengthening Telomere	PML pathway	*EME1*	Essential Meiotic Structure−Specific Endonuclease 1
Alternative Lengthening Telomere	PML pathway	*NBN*	nibrin
Alternative Lengthening Telomere	PML pathway	*MRE11A*	MRE11 homolog, double strand break repair nuclease
Alternative Lengthening Telomere	PML pathway	*RAD50*	RAD50 double strand break repair protein
Alternative Lengthening Telomere	PML pathway	*SMC5*	Structural Maintenance Of Chromosomes 5
Alternative Lengthening Telomere	PML pathway	*SMC6*	Structural Maintenance Of Chromosomes 6
Alternative Lengthening Telomere	PML pathway	*NSMCE2*	NSE2 (MMS21) Homolog, SMC5−SMC6 Complex SUMO Ligase
Alternative Lengthening Telomere	Telomere Instability	*ATRX*	ATRX Chromatin Remodeler
Alternative Lengthening Telomere	Telomere Instability	*DAXX*	Death Domain Associated Protein

Alternative Lengthening Telomere	Telomere Instability	*FEN1*	Flap Structure−Specific Endonuclease 1
Alternative Lengthening Telomere	Telomere Instability	*TERT*	telomerase reverse transcriptase
Telomerase	TERT pathway	*KPNA1*	Karyopherin Subunit Alpha 1
Telomerase	TERT pathway	*KPNB1*	Karyopherin Subunit Beta 1
Telomerase	TERT pathway	*IPO7*	Importin 7
Telomerase	TERT pathway	*HSPA1A*	Heat Shock Protein Family A (Hsp70) Member 1A
Telomerase	TERT pathway	*PTGES3*	Prostaglandin E Synthase 3
Telomerase	TERT pathway	*RANBP2*	RAN Binding Protein 2
Telomerase	TERT pathway	*XRN1*	5'−3' Exoribonuclease 1
Telomerase	TERT pathway	*DCP2*	Decapping MRNA 2
Telomerase	TERT pathway	*EXOSC3*	Exosome Component 3
Telomerase	TERT pathway	*DIS3*	DIS3 Homolog, Exosome Endoribonuclease And 3'−5' Exoribonuclease
Telomerase	TERT pathway	*PKC*	Protein Kinase C Alpha
Telomerase	TERT pathway	*ACD*	ACD shelterin complex subunit and telomerase recruitment factor
Telomerase	TERT pathway	*PINX1*	PIN2 (TERF1) Interacting Telomerase Inhibitor 1
Telomerase	TERT pathway	*TNKS1BP1*	Tankyrase 1 Binding Protein 1
Telomerase	TERT pathway	*TERF2IP*	TERF2 interacting protein
Telomerase	TERT pathway	*WRAP53*	WD Repeat Containing Antisense To TP53
Telomerase	TERT pathway	*HSP90AA1*	heat shock protein 90 alpha family class A member 1
Telomerase	TERT pathway	*HSP90AA2*	Heat Shock Protein 90 Alpha Family Class A Member 2, Pseudogene
Telomerase	TERT pathway	*HSP90AB1*	Heat Shock Protein 90 Alpha Family Class B Member 1
Telomerase	TERT pathway	*ATM*	ATM Serine/Threonine Kinase
Telomerase	TERT pathway	*HSP90*	Heat Shock Protein 90 Alpha Family Class A Member 1
Telomerase	TERT pathway	*ABL1*	ABL Proto−Oncogene 1, Non−Receptor Tyrosine Kinase
Telomerase	TERT pathway	*STUB1*	STIP1 Homology And U−Box Containing Protein 1
Telomerase	TERT pathway	*TERT*	telomerase reverse transcriptase
Telomerase	TERC_DKC1 pathway	*SRRT*	Serrate, RNA Effector Molecule
Telomerase	TERC_DKC1 pathway	*NCBP2*	Nuclear Cap Binding Protein Subunit 2
Telomerase	TERC_DKC1 pathway	*NCBP1*	Nuclear Cap Binding Protein Subunit 1
Telomerase	TERC_DKC1 pathway	*EXOSC10*	Exosome Component 10
Telomerase	TERC_DKC2 pathway	*ZCCHC7*	Zinc Finger CCHC−Type Containing 7
Telomerase	TERC_DKC1 pathway	*ZCCHC8*	Zinc Finger CCHC−Type Containing 8
Telomerase	TERC_DKC1 pathway	*MTR4*	Mtr4 Exosome RNA Helicase
Telomerase	TERC_DKC1 pathway	*PAPD5*	Terminal Nucleotidyltransferase 4B
Telomerase	TERC_DKC1 pathway	*PABPN1*	Poly(A) Binding Protein Nuclear 1
Telomerase	TERC_DKC1 pathway	*PARN*	Poly(A)−Specific Ribonuclease
Telomerase	TERC_DKC1 pathway	*FXR1*	FMR1 Autosomal Homolog 1
Telomerase	TERC_DKC1 pathway	*NAF1*	Nuclear Assembly Factor 1 Ribonucleoprotein
Telomerase	TERC_DKC1 pathway	*GAR1*	GAR1 Ribonucleoprotein
Telomerase	TERC_DKC1 pathway	*NOP10*	NOP10 Ribonucleoprotein
Telomerase	TERC_DKC1 pathway	*NHP2*	NHP2 Ribonucleoprotein
Telomerase	TERC_DKC1 pathway	*RUVBL1*	RuvB Like AAA ATPase 1
Telomerase	TERC_DKC1 pathway	*RUVBL2*	RuvB Like AAA ATPase 2
Telomerase	TERC_DKC2 pathway	*TERC*	Telomerase RNA Component
Telomerase	TERC_DKC3 pathway	*DKC1*	dyskerin pseudouridine synthase 1

Table S3. List of significant signature genes involved in the telomere maintenance mechanism across cancer types.

Cancer Type	TMM Type	Gene Name	Fold Change	*P*−value	FDR
Adrenocortical Carcinoma	Alternative Lengthening Telomere	*SMC5*	265.9212907	1.038E−06	9.028E−05
Adrenocortical Carcinoma	Alternative Lengthening Telomere	*RAD51*	89.1247	0.0001185	0.0025776
Adrenocortical Carcinoma	Alternative Lengthening Telomere	*TIMELESS*	460.3372406	0.0003262	0.0056758
Adrenocortical Carcinoma	Alternative Lengthening Telomere	*POLD4*	−486.2607043	0.0009867	0.0114623
Adrenocortical Carcinoma	Alternative Lengthening Telomere	*BRCA1*	105.4989707	0.001054	0.0114623
Adrenocortical Carcinoma	Alternative Lengthening Telomere	*RPA2*	368.8457737	0.0017021	0.0142285
Adrenocortical Carcinoma	Alternative Lengthening Telomere	*PSMC3IP*	87.08748346	0.001799	0.0142285
Adrenocortical Carcinoma	Alternative Lengthening Telomere	*RMI1*	311.4991113	0.0020533	0.0148863
Adrenocortical Carcinoma	Alternative Lengthening Telomere	*POLD3*	84.69618446	0.0029222	0.019556
Adrenocortical Carcinoma	Alternative Lengthening Telomere	*ATRX*	−387.6913128	0.0031552	0.0196071
Adrenocortical Carcinoma	Alternative Lengthening Telomere	*SUV39H2*	54.75960777	0.0057746	0.0327817
Adrenocortical Carcinoma	Alternative Lengthening Telomere	*BRCA2*	36.22815614	0.0060288	0.0327817
Adrenocortical Carcinoma	Alternative Lengthening Telomere	*PCNA*	792.9897148	0.0068429	0.0350194
Adrenocortical Carcinoma	Alternative Lengthening Telomere	*DAXX*	486.7361887	0.0095317	0.0457743
Adrenocortical Carcinoma	Alternative Lengthening Telomere	*POLD1*	222.5199967	0.0099967	0.0457743
Adrenocortical Carcinoma	Alternative Lengthening Telomere	*SUV39H1*	75.87223133	0.0119503	0.051984
Adrenocortical Carcinoma	Alternative Lengthening Telomere	*BLM*	36.93408521	0.0142248	0.0589311
Adrenocortical Carcinoma	Alternative Lengthening Telomere	*HIRA*	161.3151602	0.0157796	0.0624011
Adrenocortical Carcinoma	Alternative Lengthening Telomere	*SP100*	−306.2939063	0.0249631	0.0904913
Adrenocortical Carcinoma	Alternative Lengthening Telomere	*CHEK1*	62.77301905	0.0277434	0.0938704
Adrenocortical Carcinoma	Alternative Lengthening Telomere	*FEN1*	323.7650333	0.0280532	0.0938704
Adrenocortical Carcinoma	Alternative Lengthening Telomere	*FANCD2*	93.24558446	0.0292281	0.0941794
Adrenocortical Carcinoma	Alternative Lengthening Telomere	*EME1*	26.66690927	0.0426315	0.1278944
Adrenocortical Carcinoma	Telomerase	*NCBP1*	399.9285396	1.152E−05	0.000406
Adrenocortical Carcinoma	Telomerase	*ZCCHC7*	254.8865815	1.4E−05	0.000406
Adrenocortical Carcinoma	Telomerase	*DKC1*	368.8845965	0.0004592	0.0066582
Adrenocortical Carcinoma	Telomerase	*EXOSC3*	121.7087444	0.0015545	0.0142285
Adrenocortical Carcinoma	Telomerase	*EXOSC10*	227.093004	0.0171012	0.0646873
Adrenocortical Carcinoma	Telomerase	*KPNA1*	−311.944519	0.0341976	0.1062569
Bladder Urothelial Carcinoma	Alternative Lengthening Telomere	*RAD50*	191.6675195	0.0001021	0.0050012

Bladder Urothelial Carcinoma	Alternative Lengthening Telomere	*POLD1*	−216.1860278	0.0002507	0.0054536
Bladder Urothelial Carcinoma	Alternative Lengthening Telomere	*PSMC3IP*	−47.53978054	0.0010191	0.0132901
Bladder Urothelial Carcinoma	Alternative Lengthening Telomere	*ATRX*	189.2192729	0.0010693	0.0132901
Bladder Urothelial Carcinoma	Alternative Lengthening Telomere	*MUS81*	−97.42702787	0.001757	0.0191075
Bladder Urothelial Carcinoma	Alternative Lengthening Telomere	*POLD3*	−80.63068356	0.0021967	0.0212346
Bladder Urothelial Carcinoma	Alternative Lengthening Telomere	*FEN1*	−311.7184117	0.0030065	0.0239107
Bladder Urothelial Carcinoma	Alternative Lengthening Telomere	*HIRA*	−116.5929408	0.006418	0.0465308
Bladder Urothelial Carcinoma	Alternative Lengthening Telomere	*RPA2*	−145.0461296	0.00863	0.0577546
Bladder Urothelial Carcinoma	Alternative Lengthening Telomere	*RPA3*	−77.03893727	0.0128648	0.0799457
Bladder Urothelial Carcinoma	Alternative Lengthening Telomere	*HDAC2*	285.9562096	0.0282093	0.139961
Bladder Urothelial Carcinoma	Alternative Lengthening Telomere	*EME1*	−20.55329626	0.0433086	0.1794213
Bladder Urothelial Carcinoma	Telomerase	*PAPD5*	47.02177085	0.0001502	0.0050012
Bladder Urothelial Carcinoma	Telomerase	*RANBP2*	299.4051579	0.0001725	0.0050012
Bladder Urothelial Carcinoma	Telomerase	*ABL1*	−286.8495549	0.0009435	0.0132901
Bladder Urothelial Carcinoma	Telomerase	*HNRNPA1*	1096.768706	0.0030232	0.0239107
Bladder Urothelial Carcinoma	Telomerase	*HSP90AA1*	3488.30578	0.0173327	0.1005296
Bladder Urothelial Carcinoma	Telomerase	*RBM7*	−42.59005083	0.0275789	0.139961
Bladder Urothelial Carcinoma	Telomerase	*NHP2*	220.6072883	0.0289574	0.139961
Bladder Urothelial Carcinoma	Telomerase	*KPNA1*	122.4983032	0.031656	0.1449509
Bladder Urothelial Carcinoma	Telomerase	*XRN1*	95.70634321	0.0363688	0.1582045
Bladder Urothelial Carcinoma	Telomerase	*ZCCHC8*	47.49304619	0.0455946	0.1803058
Colon adenocarcinoma	Alternative Lengthening Telomere	*SUV39H1*	−95.91617936	6.164E−05	0.0017875
Colon adenocarcinoma	Alternative Lengthening Telomere	*RPA3*	−84.2662323	0.00265	0.0384243
Colon adenocarcinoma	Alternative Lengthening Telomere	*POT1*	−62.31632858	0.0043936	0.0424718
Colon adenocarcinoma	Alternative Lengthening Telomere	*MND1*	−27.52978962	0.0051291	0.0446236
Colon adenocarcinoma	Alternative Lengthening Telomere	*NR2C2*	85.22828505	0.0075508	0.0597198
Colon adenocarcinoma	Alternative Lengthening Telomere	*NBN*	−172.6454283	0.0125096	0.0906943
Colon adenocarcinoma	Alternative Lengthening Telomere	*SUV39H2*	−31.46163955	0.0163278	0.0972197
Colon adenocarcinoma	Alternative Lengthening Telomere	*SMC5*	−86.0081524	0.0179827	0.0972197
Colon adenocarcinoma	Alternative Lengthening Telomere	*DAXX*	104.9446452	0.0199727	0.0972197
Colon adenocarcinoma	Alternative Lengthening Telomere	*DKC1*	−368.5231456	0.0201144	0.0972197
Colon adenocarcinoma	Alternative Lengthening Telomere	*RAD51*	−43.76105416	0.0284806	0.112628

Colon adenocarcinoma	Telomerase	*PTGES3*	−1302.307437	1.299E−05	0.0008741
Colon adenocarcinoma	Telomerase	*ABL1*	344.3948538	2.01E−05	0.0008741
Colon adenocarcinoma	Telomerase	*RMI1*	−52.6496001	0.0017012	0.0370013
Colon adenocarcinoma	Telomerase	*DCP2*	65.02824065	0.0023777	0.0384243
Colon adenocarcinoma	Telomerase	*NSMCE2*	−65.48236096	0.0041543	0.0424718
Colon adenocarcinoma	Telomerase	*NCBP1*	−142.172615	0.0043326	0.0424718
Colon adenocarcinoma	Telomerase	*NHP2*	−246.3501179	0.0153269	0.0972197
Colon adenocarcinoma	Telomerase	*TIPIN*	−18.18215105	0.0185731	0.0972197
Colon adenocarcinoma	Telomerase	*NAF1*	−17.09019109	0.0233412	0.1068784
Colon adenocarcinoma	Telomerase	*HSP90AA1*	−3111.267912	0.0260933	0.112628
Colon adenocarcinoma	Telomerase	*NOP10*	−142.824519	0.027431	0.112628
Colon adenocarcinoma	Telomerase	*DIS3*	−126.0299915	0.0322218	0.1218826
Colon adenocarcinoma	Telomerase	*TERT*	9.682226984	0.0342115	0.1240166
Lymphoid Neoplasm Diffuse Large B−cell Lymphoma	Alternative Lengthening Telomere	*HDAC9*	323.8733398	0.0350719	0.9845744
Lymphoid Neoplasm Diffuse Large B−cell Lymphoma	Telomerase	*DCP2*	412.8499799	0.033982	0.9845744
Esophageal carcinoma	Alternative Lengthening Telomere	*BLM*	−99.17838491	0.0012367	0.1075895
Esophageal carcinoma	Alternative Lengthening Telomere	*BRCA1*	−239.472868	0.0050093	0.1124741
Esophageal carcinoma	Alternative Lengthening Telomere	*SMC6*	−257.4652559	0.0075612	0.1124741
Esophageal carcinoma	Alternative Lengthening Telomere	*TIMELESS*	−251.0314195	0.0090496	0.1124741
Esophageal carcinoma	Alternative Lengthening Telomere	*RAD51*	−56.96295255	0.0154228	0.1571964
Esophageal carcinoma	Alternative Lengthening Telomere	*EME1*	−27.6486921	0.0170077	0.1571964
Esophageal carcinoma	Alternative Lengthening Telomere	*NR2F2*	807.3399005	0.0276767	0.2188972
Esophageal carcinoma	Alternative Lengthening Telomere	*SUV39H1*	−68.77434709	0.039847	0.2707443
Esophageal carcinoma	Telomerase	*GEN1*	−150.978332	0.0058863	0.1124741
Esophageal carcinoma	Telomerase	*RNASEH1*	−44.64417768	0.0073327	0.1124741
Esophageal carcinoma	Telomerase	*NSMCE2*	67.86429568	0.0082619	0.1124741
Esophageal carcinoma	Telomerase	*HSP90AA1*	−4822.87089	0.0180686	0.1571964
Esophageal carcinoma	Telomerase	*CBX5*	−796.8697181	0.040456	0.2707443
Glioblastoma multiforme	Alternative Lengthening Telomere	*SUV39H2*	−42.22909714	0.0208871	0.6353327
Glioblastoma multiforme	Telomerase	*GAR1*	33.52825826	0.0153689	0.6353327

Glioblastoma multiforme	Telomerase	*PTGES3*	−727.8328605	0.0345896	0.6353327
Glioblastoma multiforme	Telomerase	*PARN*	81.81379438	0.0372365	0.6353327
Glioblastoma multiforme	Telomerase	*KPNB1*	401.3272102	0.0408458	0.6353327
Head and Neck squamous cell carcinoma	Alternative Lengthening Telomere	*BRCA1*	−78.65842301	0.0067657	0.5886193
Head and Neck squamous cell carcinoma	Alternative Lengthening Telomere	*SMC5*	81.07664145	0.0291933	0.7703089
Head and Neck squamous cell carcinoma	Alternative Lengthening Telomere	*RAD51*	−38.8564133	0.0466194	0.811178
Head and Neck squamous cell carcinoma	Telomerase	*ABL1*	180.4515059	0.0299859	0.7703089
Head and Neck squamous cell carcinoma	Telomerase	*KPNB1*	−335.3412803	0.0354165	0.7703089
Kidney Chromophobe	Alternative Lengthening Telomere	*FANCD2*	83.4806633	0.0073204	0.1273741
Kidney Chromophobe	Alternative Lengthening Telomere	*WRN*	231.9415106	0.0089846	0.1302774
Kidney Chromophobe	Alternative Lengthening Telomere	*RFC1*	396.6385044	0.0104847	0.1303096
Kidney Chromophobe	Alternative Lengthening Telomere	*POT1*	182.7564113	0.0194534	0.2017912
Kidney Chromophobe	Alternative Lengthening Telomere	*MND1*	27.29443424	0.020875	0.2017912
Kidney Chromophobe	Alternative Lengthening Telomere	*DAXX*	−160.5915732	0.0328684	0.285955
Kidney Chromophobe	Alternative Lengthening Telomere	*RAD50*	598.943452	0.0427888	0.3384203
Kidney Chromophobe	Telomerase	*RBM7*	214.4184665	0.0011499	0.1000438
Kidney Chromophobe	Telomerase	*CBX5*	1023.144951	0.0032399	0.1235664
Kidney Chromophobe	Telomerase	*NCBP1*	232.7781978	0.0042609	0.1235664
Kidney Chromophobe	Telomerase	*TERT*	294.8574874	0.0062861	0.1273741
Kidney renal clear cell carcinoma	Alternative Lengthening Telomere	*WRN*	30.51488522	0.0099325	0.4287012
Kidney renal clear cell carcinoma	Alternative Lengthening Telomere	*AKTIP*	93.26949482	0.0147828	0.4287012
Kidney renal clear cell carcinoma	Alternative Lengthening Telomere	*MRE11A*	31.44136993	0.0369851	0.5287595
Kidney renal clear cell carcinoma	Alternative Lengthening Telomere	*ATRX*	178.9262485	0.0371978	0.5287595
Kidney renal clear cell carcinoma	Alternative Lengthening Telomere	*RAD50*	169.6982295	0.0425439	0.5287595
Kidney renal clear cell carcinoma	Telomerase	*NCBP1*	68.40541455	0.0033802	0.2940805
Kidney renal clear cell carcinoma	Telomerase	*ZCCHC7*	35.69440563	0.0263797	0.5287595
Kidney renal clear cell carcinoma	Telomerase	*NHP2*	−160.3835171	0.0487888	0.5305785
Kidney renal papillary cell carcinoma	Alternative Lengthening Telomere	*ATR*	−93.26737592	0.0311272	0.9895902

Kidney renal papillary cell carcinoma	Telomerase	*SRRT*	−249.2372547	0.0170805	0.9895902
Acute Myeloid Leukemia	Alternative Lengthening Telomere	*EME1*	−32.35781584	0.0417124	0.6282464
Acute Myeloid Leukemia	Alternative Lengthening Telomere	*POT1*	−61.61096958	0.0436787	0.6282464
Acute Myeloid Leukemia	Telomerase	*ZCCHC8*	−49.59917446	0.0422015	0.6282464
Brain Lower Grade Glioma	Alternative Lengthening Telomere	*ATRX*	−609.694306	1.61E−11	1.4E−09
Brain Lower Grade Glioma	Alternative Lengthening Telomere	*HDAC1*	186.6615676	2.46E−07	1.07E−05
Brain Lower Grade Glioma	Alternative Lengthening Telomere	*RPA2*	144.4174022	1.506E−06	3.276E−05
Brain Lower Grade Glioma	Alternative Lengthening Telomere	*SUV39H2*	28.49744615	3.932E−05	0.0006841
Brain Lower Grade Glioma	Alternative Lengthening Telomere	*TERT*	−4.366824531	6.618E−05	0.0009597
Brain Lower Grade Glioma	Alternative Lengthening Telomere	*HDAC9*	−106.1952031	0.0001266	0.0013826
Brain Lower Grade Glioma	Alternative Lengthening Telomere	*SUV39H1*	−30.99584809	0.0001271	0.0013826
Brain Lower Grade Glioma	Alternative Lengthening Telomere	*PML*	−115.9932158	0.000211	0.00204
Brain Lower Grade Glioma	Alternative Lengthening Telomere	*BLM*	−103.9467439	0.0003948	0.0034348
Brain Lower Grade Glioma	Alternative Lengthening Telomere	*NBN*	111.6589672	0.0011501	0.0076965
Brain Lower Grade Glioma	Alternative Lengthening Telomere	*SMC5*	75.83544044	0.0029388	0.0182623
Brain Lower Grade Glioma	Alternative Lengthening Telomere	*AKTIP*	72.54987381	0.004416	0.0256129
Brain Lower Grade Glioma	Alternative Lengthening Telomere	*RFC1*	76.8813009	0.0048237	0.0262289
Brain Lower Grade Glioma	Alternative Lengthening Telomere	*RAD50*	−55.66962603	0.0267526	0.1223064
Brain Lower Grade Glioma	Alternative Lengthening Telomere	*RPA1*	91.73625583	0.0458096	0.1689393
Brain Lower Grade Glioma	Telomerase	*EXOSC10*	191.4204841	3.822E−07	1.108E−05
Brain Lower Grade Glioma	Telomerase	*ABL1*	276.0061839	0.0005842	0.0046203
Brain Lower Grade Glioma	Telomerase	*NCBP1*	75.9825115	0.0007068	0.0051244
Brain Lower Grade Glioma	Telomerase	*HSP90AB1*	−1379.384814	0.0080316	0.0411031
Brain Lower Grade Glioma	Telomerase	*RUVBL2*	115.8204691	0.0202727	0.0979846
Brain Lower Grade Glioma	Telomerase	*RBM7*	22.05837516	0.0281164	0.1223064
Brain Lower Grade Glioma	Telomerase	*PARN*	31.18941665	0.035031	0.1451283
Brain Lower Grade Glioma	Telomerase	*RANBP2*	91.16249125	0.0447669	0.1689393
Brain Lower Grade Glioma	Telomerase	*NHP2*	−88.05635692	0.046604	0.1689393
Liver hepatocellular carcinoma	Alternative Lengthening Telomere	*HDAC2*	334.2247182	1.821E−05	0.0015845
Liver hepatocellular carcinoma	Alternative Lengthening Telomere	*SMC5*	142.1207619	0.0002092	0.0060674

Liver hepatocellular carcinoma	Alternative Lengthening Telomere	*RFC1*	352.1383427	0.0003935	0.0085583
Liver hepatocellular carcinoma	Alternative Lengthening Telomere	*PML*	−231.1475954	0.0015702	0.0202268
Liver hepatocellular carcinoma	Alternative Lengthening Telomere	*FANCD2*	82.78926157	0.0016274	0.0202268
Liver hepatocellular carcinoma	Alternative Lengthening Telomere	*POLD1*	169.5315527	0.0024524	0.0266693
Liver hepatocellular carcinoma	Alternative Lengthening Telomere	*MRE11A*	45.5937519	0.0042317	0.0409062
Liver hepatocellular carcinoma	Alternative Lengthening Telomere	*WRN*	40.19410781	0.0050485	0.0439218
Liver hepatocellular carcinoma	Alternative Lengthening Telomere	*BRCA2*	20.14668508	0.0112405	0.0752248
Liver hepatocellular carcinoma	Alternative Lengthening Telomere	*TIMELESS*	189.5702109	0.0128229	0.0796849
Liver hepatocellular carcinoma	Alternative Lengthening Telomere	*EME1*	23.34240227	0.0165356	0.0884086
Liver hepatocellular carcinoma	Alternative Lengthening Telomere	*SP100*	−196.058832	0.0179362	0.0884086
Liver hepatocellular carcinoma	Alternative Lengthening Telomere	*DKC1*	161.5160461	0.0279127	0.1278106
Liver hepatocellular carcinoma	Alternative Lengthening Telomere	*BRCA1*	52.09747929	0.0324409	0.1411179
Liver hepatocellular carcinoma	Alternative Lengthening Telomere	*BLM*	27.41594983	0.0362022	0.1431634
Liver hepatocellular carcinoma	Alternative Lengthening Telomere	*SUV39H2*	23.28109097	0.0466566	0.1730926
Liver hepatocellular carcinoma	Alternative Lengthening Telomere	*RAD51*	23.01926389	0.0477497	0.1730926
Liver hepatocellular carcinoma	Telomerase	*NCBP1*	118.937451	0.0001065	0.0046338
Liver hepatocellular carcinoma	Telomerase	*FXR1*	317.4245141	0.0005734	0.0099771
Liver hepatocellular carcinoma	Telomerase	*NCBP2*	174.4383168	0.006012	0.0475492
Liver hepatocellular carcinoma	Telomerase	*EXOSC10*	128.6618364	0.0074311	0.0538757
Liver hepatocellular carcinoma	Telomerase	*EXOSC3*	42.24098313	0.0147004	0.0852625
Liver hepatocellular carcinoma	Telomerase	*ZCCHC7*	35.90525503	0.0182914	0.0884086

Liver hepatocellular carcinoma	Telomerase	*DIS3*	72.34635423	0.0341816	0.1416096
Lung adenocarcinoma	Alternative Lengthening Telomere	*RPA1*	132.4162373	0.011612	0.4856134
Lung adenocarcinoma	Alternative Lengthening Telomere	*BLM*	31.93709087	0.0193783	0.4856134
Lung adenocarcinoma	Alternative Lengthening Telomere	*WRN*	23.90000506	0.0491469	0.6099617
Lung adenocarcinoma	Telomerase	*DCP2*	70.26246338	0.0107659	0.4856134
Lung adenocarcinoma	Telomerase	*TERT*	13.77944266	0.0223271	0.4856134
Lung adenocarcinoma	Telomerase	*NCBP1*	58.88605638	0.0329591	0.5064675
Lung adenocarcinoma	Telomerase	*WRAP53*	22.15918273	0.0349288	0.5064675
Lung squamous cell carcinoma	Alternative Lengthening Telomere	*RNASEH1*	−40.65247512	0.0102974	0.7414902
Lung squamous cell carcinoma	Alternative Lengthening Telomere	*BRCA2*	−25.15859555	0.0241107	0.7414902
Lung squamous cell carcinoma	Alternative Lengthening Telomere	*SMC6*	−141.1723628	0.0255686	0.7414902
Ovarian serous cystadenocarcinoma	Alternative Lengthening Telomere	*ATRX*	1139.785101	0.0021564	0.1876071
Ovarian serous cystadenocarcinoma	Alternative Lengthening Telomere	*ZNF827*	327.9467405	0.0232657	0.5451416
Ovarian serous cystadenocarcinoma	Alternative Lengthening Telomere	*TOP3A*	120.1311076	0.025064	0.5451416
Ovarian serous cystadenocarcinoma	Alternative Lengthening Telomere	*NR2C2*	447.3485748	0.0469589	0.6775091
Ovarian serous cystadenocarcinoma	Telomerase	*DIS3*	350.0966086	0.0081621	0.3550495
Ovarian serous cystadenocarcinoma	Telomerase	*SRRT*	382.3369433	0.037868	0.6589041
Pancreatic adenocarcinoma	Alternative Lengthening Telomere	*RFC1*	179.6269131	0.0004911	0.0427284
Pancreatic adenocarcinoma	Alternative Lengthening Telomere	*NBN*	168.7009007	0.0034449	0.1498519
Pancreatic adenocarcinoma	Alternative Lengthening Telomere	*WRN*	48.91903537	0.005169	0.1499005
Pancreatic adenocarcinoma	Alternative Lengthening Telomere	*RAD52*	−30.83556251	0.0091589	0.1992068
Pancreatic adenocarcinoma	Alternative Lengthening Telomere	*FEN1*	97.47655918	0.0125992	0.2027396
Pancreatic adenocarcinoma	Alternative Lengthening Telomere	*TOP3A*	52.11143346	0.0168821	0.2098206
Pancreatic adenocarcinoma	Alternative Lengthening Telomere	*POLD3*	71.78929039	0.0208552	0.2268004
Pancreatic adenocarcinoma	Alternative Lengthening Telomere	*HIRA*	73.59774459	0.0243737	0.2356124
Pancreatic adenocarcinoma	Alternative Lengthening Telomere	*RPA1*	136.8478254	0.0346327	0.2983155
Pancreatic adenocarcinoma	Alternative Lengthening Telomere	*SUV39H1*	27.44894509	0.0377181	0.2983155
Pancreatic adenocarcinoma	Telomerase	*XRN1*	143.1846621	0.013982	0.2027396

Pancreatic adenocarcinoma	Telomerase	*HSPA1A*	−14074.41479	0.0465545	0.3375198
Pheochromocytoma and Paraganglioma	Alternative Lengthening Telomere	*NR2F2*	−256.9306438	0.0161019	0.7389156
Pheochromocytoma and Paraganglioma	Telomerase	*DCP2*	−71.5758424	0.0169866	0.7389156
Pheochromocytoma and Paraganglioma	Telomerase	*RANBP2*	1236.960993	0.0424614	0.9745967
Pheochromocytoma and Paraganglioma	Telomerase	*NAF1*	23.07205923	0.0450306	0.9745967
Prostate adenocarcinoma	Alternative Lengthening Telomere	*POT1*	−52.35242353	0.0237522	0.4402657
Prostate adenocarcinoma	Alternative Lengthening Telomere	*POLD1*	27.47492714	0.0357694	0.4402657
Prostate adenocarcinoma	Alternative Lengthening Telomere	*FANCD2*	−17.87870212	0.0416027	0.4402657
Prostate adenocarcinoma	Alternative Lengthening Telomere	*SUV39H2*	−32.34443476	0.0432153	0.4402657
Prostate adenocarcinoma	Telomerase	*IPO7*	−342.1024282	0.0076963	0.4402657
Prostate adenocarcinoma	Telomerase	*KPNB1*	−339.4113665	0.0121801	0.4402657
Prostate adenocarcinoma	Telomerase	*PAPD5*	−31.65754678	0.0242979	0.4402657
Prostate adenocarcinoma	Telomerase	*RBM7*	47.28687862	0.0437345	0.4402657
Prostate adenocarcinoma	Telomerase	*NSMCE2*	−25.37165544	0.0455447	0.4402657
Sarcoma	Alternative Lengthening Telomere	*SUV39H1*	163.8847432	1.364E−05	0.0011864
Sarcoma	Alternative Lengthening Telomere	*TOP3A*	412.6583071	3.898E−05	0.0016955
Sarcoma	Alternative Lengthening Telomere	*PSMC3IP*	113.77488	6.699E−05	0.0019427
Sarcoma	Alternative Lengthening Telomere	*HNRNPA1*	−2064.374385	0.0003519	0.0058351
Sarcoma	Alternative Lengthening Telomere	*SMARCAL1*	−94.34847449	0.0004024	0.0058351
Sarcoma	Alternative Lengthening Telomere	*BRCA1*	104.0050334	0.0006985	0.0082037
Sarcoma	Alternative Lengthening Telomere	*RAD51*	74.5201408	0.0007544	0.0082037
Sarcoma	Alternative Lengthening Telomere	*EME1*	40.64631006	0.001361	0.0131567
Sarcoma	Alternative Lengthening Telomere	*FEN1*	337.430963	0.0016142	0.0140439
Sarcoma	Alternative Lengthening Telomere	*ZNF827*	−63.56617094	0.0023821	0.01884
Sarcoma	Alternative Lengthening Telomere	*RPA1*	459.1310821	0.0027538	0.019965
Sarcoma	Alternative Lengthening Telomere	*MND1*	30.0919347	0.0047155	0.0315576
Sarcoma	Alternative Lengthening Telomere	*FANCD2*	91.24536158	0.006351	0.0394667
Sarcoma	Alternative Lengthening Telomere	*PCNA*	532.1228774	0.0085695	0.0486198
Sarcoma	Alternative Lengthening Telomere	*BLM*	37.77321508	0.0090637	0.0486198
Sarcoma	Alternative Lengthening Telomere	*HIRA*	141.8507564	0.0095004	0.0486198

Sarcoma	Alternative Lengthening Telomere	*HSP90AB1*	−5098.793686	0.0121078	0.0585209
Sarcoma	Alternative Lengthening Telomere	*HSPA1A*	8751.706317	0.0184763	0.07767
Sarcoma	Alternative Lengthening Telomere	*POLD3*	142.4680738	0.0187479	0.07767
Sarcoma	Alternative Lengthening Telomere	*RMI1*	67.70615016	0.0206147	0.0798162
Sarcoma	Alternative Lengthening Telomere	*BRCA2*	42.9271681	0.0273387	0.0991027
Sarcoma	Alternative Lengthening Telomere	*RPA2*	159.9495441	0.028912	0.1006138
Sarcoma	Alternative Lengthening Telomere	*MUS81*	111.6458426	0.0335945	0.1076203
Sarcoma	Alternative Lengthening Telomere	*ATR*	−53.8095434	0.0351428	0.1076203
Sarcoma	Alternative Lengthening Telomere	*RFC1*	168.328652	0.0358734	0.1076203
Sarcoma	Telomerase	*NCBP2*	−243.622948	0.0001891	0.004113
Sarcoma	Telomerase	*DCP2*	−117.8422808	0.0143645	0.0657741
Sarcoma	Telomerase	*EXOSC10*	162.0355077	0.0211008	0.0798162
Sarcoma	Telomerase	*NAF1*	−17.12668947	0.0329876	0.1076203
Sarcoma	Telomerase	*KPNA1*	184.3383016	0.0374548	0.108619
Sarcoma	Telomerase	*DIS3*	−85.48296084	0.0392038	0.1100235
Skin Cutaneous Melanoma	Alternative Lengthening Telomere	*BRCA2*	98.93527143	0.0005717	0.0497376
Skin Cutaneous Melanoma	Alternative Lengthening Telomere	*BLM*	137.9910377	0.0141384	0.2691104
Skin Cutaneous Melanoma	Alternative Lengthening Telomere	*FEN1*	551.2647792	0.0160333	0.2691104
Skin Cutaneous Melanoma	Alternative Lengthening Telomere	*RAD50*	279.2731623	0.0177715	0.2691104
Skin Cutaneous Melanoma	Alternative Lengthening Telomere	*NBN*	295.8064942	0.0376064	0.418398
Skin Cutaneous Melanoma	Alternative Lengthening Telomere	*TIMELESS*	208.8230952	0.0450721	0.4356973
Skin Cutaneous Melanoma	Telomerase	*IPO7*	996.8992713	0.0139074	0.2691104
Skin Cutaneous Melanoma	Telomerase	*NCBP1*	261.1547616	0.0185593	0.2691104
Skin Cutaneous Melanoma	Telomerase	*RANBP2*	436.1832472	0.0384734	0.418398
Stomach adenocarcinoma	Alternative Lengthening Telomere	*ATR*	−144.2373981	0.0014448	0.1256965
Stomach adenocarcinoma	Alternative Lengthening Telomere	*BRCA1*	−110.3838056	0.0073689	0.3205473
Stomach adenocarcinoma	Telomerase	*XRN1*	−153.9415303	0.0215248	0.6242195
Stomach adenocarcinoma	Telomerase	*RUVBL2*	191.4352574	0.0492138	0.7642745
Testicular Germ Cell Tumors	Alternative Lengthening Telomere	*RPA1*	−1099.863178	1.003E−11	6.657E−10
Testicular Germ Cell Tumors	Alternative Lengthening Telomere	*HDAC1*	1610.980592	1.315E−10	2.86E−09
Testicular Germ Cell Tumors	Alternative Lengthening Telomere	*RANBP2*	−2032.631737	2.96E−10	5.151E−09
Testicular Germ Cell Tumors	Alternative Lengthening Telomere	*HDAC9*	−518.4795547	3.769E−10	5.464E−09
Testicular Germ Cell Tumors	Alternative Lengthening Telomere	*ATR*	−183.5832718	7.234E−09	8.991E−08
Testicular Germ Cell Tumors	Alternative Lengthening Telomere	*MUS81*	−222.7034843	1.033E−08	1.124E−07
Testicular Germ Cell Tumors	Alternative Lengthening Telomere	*NBN*	−474.2268304	2.334E−08	2.256E−07

Testicular Germ Cell Tumors	Alternative Lengthening Telomere	*TIMELESS*	−1241.797273	7.954E−08	6.92E−07
Testicular Germ Cell Tumors	Alternative Lengthening Telomere	*RAD51*	−563.7778129	1.205E−07	9.534E−07
Testicular Germ Cell Tumors	Alternative Lengthening Telomere	*POT1*	−187.6328458	1.99E−07	1.443E−06
Testicular Germ Cell Tumors	Alternative Lengthening Telomere	*RMI1*	−167.9946816	3.363E−07	2.25E−06
Testicular Germ Cell Tumors	Alternative Lengthening Telomere	*TIPIN*	−136.6755756	8.274E−07	4.799E−06
Testicular Germ Cell Tumors	Alternative Lengthening Telomere	*NR2C2*	−350.1467738	6.378E−06	2.774E−05
Testicular Germ Cell Tumors	Alternative Lengthening Telomere	*WRN*	−304.8324998	8.434E−06	3.494E−05
Testicular Germ Cell Tumors	Alternative Lengthening Telomere	*FANCD2*	−511.8438694	1.004E−05	3.796E−05
Testicular Germ Cell Tumors	Alternative Lengthening Telomere	*BLM*	−263.1245241	1.735E−05	6.038E−05
Testicular Germ Cell Tumors	Alternative Lengthening Telomere	*CBX5*	1596.618186	3.888E−05	0.0001253
Testicular Germ Cell Tumors	Alternative Lengthening Telomere	*BRCA2*	−86.08212932	0.0001113	0.0003458
Testicular Germ Cell Tumors	Alternative Lengthening Telomere	*GEN1*	−209.1105648	0.0001796	0.0005208
Testicular Germ Cell Tumors	Alternative Lengthening Telomere	*MND1*	−95.82791083	0.0006684	0.001762
Testicular Germ Cell Tumors	Alternative Lengthening Telomere	*HDAC2*	771.7230819	0.0011921	0.0030504
Testicular Germ Cell Tumors	Alternative Lengthening Telomere	*NR2F2*	537.896994	0.0016955	0.0042144
Testicular Germ Cell Tumors	Alternative Lengthening Telomere	*CHEK1*	−164.1052328	0.0018601	0.0044952
Testicular Germ Cell Tumors	Alternative Lengthening Telomere	*HNRNPA1*	2603.208124	0.0027064	0.0063638
Testicular Germ Cell Tumors	Alternative Lengthening Telomere	*BRCA1*	−250.5550674	0.0040535	0.0092804
Testicular Germ Cell Tumors	Alternative Lengthening Telomere	*ZNF827*	49.95220914	0.0045248	0.0098415
Testicular Germ Cell Tumors	Alternative Lengthening Telomere	*MRE11A*	−116.3656577	0.0050227	0.010658
Testicular Germ Cell Tumors	Alternative Lengthening Telomere	*POLD3*	−85.21227611	0.0088169	0.0182635
Testicular Germ Cell Tumors	Alternative Lengthening Telomere	*SUV39H2*	−40.44804594	0.0114051	0.0230755
Testicular Germ Cell Tumors	Alternative Lengthening Telomere	*RPA3*	147.0547478	0.0155875	0.0308208
Testicular Germ Cell Tumors	Alternative Lengthening Telomere	*SMC6*	−144.3416986	0.0258072	0.0477707
Testicular Germ Cell Tumors	Alternative Lengthening Telomere	*RFC1*	−141.7450551	0.0295344	0.0530493
Testicular Germ Cell Tumors	Alternative Lengthening Telomere	*DAXX*	−200.890387	0.0437836	0.0732144
Testicular Germ Cell Tumors	Telomerase	*DCP2*	−1138.887701	1.53E−11	6.657E−10
Testicular Germ Cell Tumors	Telomerase	*RBM7*	218.6133244	7.971E−11	2.312E−09
Testicular Germ Cell Tumors	Telomerase	*XRN1*	−280.9464329	6.626E−07	4.118E−06
Testicular Germ Cell Tumors	Telomerase	*WRAP53*	185.1535024	1.49E−06	8.1E−06
Testicular Germ Cell Tumors	Telomerase	*ZCCHC7*	−190.2781813	1.809E−06	9.259E−06
Testicular Germ Cell Tumors	Telomerase	*DIS3*	−186.2629535	4.814E−06	2.327E−05

Testicular Germ Cell Tumors	Telomerase	*DKC1*	953.3485971	6.087E−06	2.774E−05
Testicular Germ Cell Tumors	Telomerase	*TERT*	76.0103736	8.891E−06	3.516E−05
Testicular Germ Cell Tumors	Telomerase	*PAPD5*	−156.3177048	1.05E−05	3.807E−05
Testicular Germ Cell Tumors	Telomerase	*GAR1*	137.3491475	3.585E−05	0.0001199
Testicular Germ Cell Tumors	Telomerase	*STUB1*	366.486603	0.0001416	0.0004248
Testicular Germ Cell Tumors	Telomerase	*NSMCE2*	56.8616138	0.0002143	0.0006015
Testicular Germ Cell Tumors	Telomerase	*ZCCHC8*	−167.6366265	0.0004094	0.001113
Testicular Germ Cell Tumors	Telomerase	*HSP90AA1*	−11260.49093	0.004368	0.0097439
Testicular Germ Cell Tumors	Telomerase	*NHP2*	307.6138429	0.0200229	0.0387109
Testicular Germ Cell Tumors	Telomerase	*NAF1*	−36.4430379	0.025036	0.0473506
Testicular Germ Cell Tumors	Telomerase	*HSP90AB1*	−8153.115873	0.0298784	0.0530493
Testicular Germ Cell Tumors	Telomerase	*IPO7*	538.5722565	0.031263	0.0543977
Testicular Germ Cell Tumors	Telomerase	*EXOSC3*	84.36967288	0.0382849	0.0653096
Testicular Germ Cell Tumors	Telomerase	*HSPA1A*	3515.562008	0.0446019	0.0732144
Testicular Germ Cell Tumors	Telomerase	*ABL1*	−353.7219804	0.0496503	0.0799922
Thyroid carcinoma	Alternative Lengthening Telomere	*POLD1*	−58.02157718	0.0053032	0.2306902
Thyroid carcinoma	Telomerase	*WRAP53*	−15.53232982	0.0241791	0.7011949
Thyroid carcinoma	Telomerase	*NSMCE2*	16.28303387	0.0347787	0.7564367
Thyroid carcinoma	Telomerase	*SRRT*	−98.94196188	0.00281	0.2306902
Thymoma	Alternative Lengthening Telomere	*MND1*	186.0696751	6.932E−06	0.000434
Thymoma	Alternative Lengthening Telomere	*BLM*	202.4075155	1.566E−05	0.000454
Thymoma	Alternative Lengthening Telomere	*HDAC1*	924.0057188	2.128E−05	0.0004629
Thymoma	Alternative Lengthening Telomere	*RAD51*	208.6873481	3.747E−05	0.0005389
Thymoma	Alternative Lengthening Telomere	*HNRNPA1*	4491.091792	4.361E−05	0.0005389
Thymoma	Alternative Lengthening Telomere	*SUV39H1*	220.162048	4.436E−05	0.0005389
Thymoma	Alternative Lengthening Telomere	*CHEK1*	178.7347174	5.197E−05	0.0005389
Thymoma	Alternative Lengthening Telomere	*PCNA*	2068.607978	5.876E−05	0.0005389
Thymoma	Alternative Lengthening Telomere	*FEN1*	724.7548377	6.469E−05	0.0005389
Thymoma	Alternative Lengthening Telomere	*DAXX*	430.6437697	6.922E−05	0.0005389
Thymoma	Alternative Lengthening Telomere	*MUS81*	191.6097782	7.434E−05	0.0005389
Thymoma	Alternative Lengthening Telomere	*NBN*	−259.1699586	0.0001517	0.0010155
Thymoma	Alternative Lengthening Telomere	*RPA3*	257.0866531	0.0004467	0.0022858

Thymoma	Alternative Lengthening Telomere	*HSP90AB1*	−5244.963214	0.0005211	0.0025186
Thymoma	Alternative Lengthening Telomere	*RAD50*	−253.9567146	0.0009038	0.0041386
Thymoma	Alternative Lengthening Telomere	*HDAC2*	490.786445	0.0016347	0.0070181
Thymoma	Alternative Lengthening Telomere	*POLD1*	474.7586696	0.0048752	0.0176726
Thymoma	Alternative Lengthening Telomere	*FANCD2*	128.3363825	0.0063759	0.0211499
Thymoma	Alternative Lengthening Telomere	*SUV39H2*	46.77178456	0.0066292	0.0211499
Thymoma	Alternative Lengthening Telomere	*TIPIN*	28.89160172	0.0068069	0.0211499
Thymoma	Alternative Lengthening Telomere	*EME1*	37.88500441	0.0072401	0.0217202
Thymoma	Alternative Lengthening Telomere	*NR2F2*	−382.8986286	0.0075787	0.0219783
Thymoma	Alternative Lengthening Telomere	*HIRA*	193.8543609	0.0140105	0.0338588
Thymoma	Alternative Lengthening Telomere	*TIMELESS*	244.1776894	0.0153484	0.0351859
Thymoma	Alternative Lengthening Telomere	*ZNF827*	−50.72725499	0.0163803	0.0365407
Thymoma	Alternative Lengthening Telomere	*ATR*	−94.6983106	0.0191357	0.0416202
Thymoma	Alternative Lengthening Telomere	*SP100*	−325.9184468	0.0201606	0.0427799
Thymoma	Alternative Lengthening Telomere	*RAD52*	−32.02281382	0.030866	0.0639367
Thymoma	Alternative Lengthening Telomere	*RUVBL1*	225.8888906	0.0370234	0.0749078
Thymoma	Alternative Lengthening Telomere	*BRCA1*	78.43731605	0.0402997	0.0788794
Thymoma	Telomerase	*DCP2*	628.1239946	9.977E−06	0.000434
Thymoma	Telomerase	*NSMCE2*	80.24766406	0.0003299	0.0020502
Thymoma	Telomerase	*EXOSC3*	103.1819738	0.0003654	0.0020696
Thymoma	Telomerase	*SRRT*	567.44101	0.0003806	0.0020696
Thymoma	Telomerase	*GEN1*	81.90362077	0.001694	0.0070181
Thymoma	Telomerase	*GAR1*	114.3864968	0.0018312	0.0072416
Thymoma	Telomerase	*NOP10*	513.9592536	0.0023308	0.0088166
Thymoma	Telomerase	*NAF1*	−30.93893793	0.0060098	0.0209141
Thymoma	Telomerase	*IPO7*	−392.7189627	0.0082738	0.0230416
Thymoma	Telomerase	*WRAP53*	185.8216997	0.0087092	0.0230416
Thymoma	Telomerase	*RUVBL2*	747.6621579	0.0087399	0.0230416
Thymoma	Telomerase	*HSPA1A*	−3252.026293	0.0099146	0.0253696
Thymoma	Telomerase	*ABL1*	−492.6940095	0.0129565	0.0322061
Thymoma	Telomerase	*TERT*	123.0246944	0.0153685	0.0351859
Thymoma	Telomerase	*DIS3*	−89.20213722	0.0407997	0.0788794
Uterine Corpus Endometrial Carcinoma	Alternative Lengthening Telomere	*MND1*	175.53818	0.0454705	0.9427162
Uterine Corpus Endometrial Carcinoma	Alternative Lengthening Telomere	*SP100*	−601.1515133	0.0478386	0.9427162
Uterine Corpus Endometrial Carcinoma	Telomerase	*EXOSC10*	410.79996	0.0295713	0.9427162
Uterine Corpus Endometrial Carcinoma	Telomerase	*DCP2*	−430.23276	0.0415873	0.9427162

Table S4. List of significant gene ontology (GO) terms enriched in the high ALT and low ALT groups.

Cancer Types	ALT Group	Survival Group	Category	Term	Description	LogP	Log(q−value)
Breast invasive carcinoma, Sarcoma, Lung adenocarcinoma	High	Poor	Reactome Gene Sets	R−HSA−1640170	Cell Cycle	−100	−95.682459
Breast invasive carcinoma, Sarcoma, Lung adenocarcinoma	High	Poor	GO Biological Processes	GO:0006281	DNA repair	−78.305	−74.4648032
Breast invasive carcinoma, Sarcoma, Lung adenocarcinoma	High	Poor	GO Biological Processes	GO:0006260	DNA replication	−77.512	−73.7967916
Breast invasive carcinoma, Sarcoma, Lung adenocarcinoma	High	Poor	GO Biological Processes	GO:0007059	chromosome segregation	−64.451	−60.8326314
Breast invasive carcinoma, Sarcoma, Lung adenocarcinoma	High	Poor	GO Biological Processes	GO:0010564	regulation of cell cycle process	−55.749	−52.3857411
Breast invasive carcinoma, Sarcoma, Lung adenocarcinoma	High	Poor	GO Biological Processes	GO:0051052	regulation of DNA metabolic process	−48.714	−45.6003623
Breast invasive carcinoma, Sarcoma, Lung adenocarcinoma	High	Poor	GO Biological Processes	GO:0007017	microtubule−based process	−46.215	−43.1985846
Breast invasive carcinoma, Sarcoma, Lung adenocarcinoma	High	Poor	Reactome Gene Sets	R−HSA−73894	DNA Repair	−45.732	−42.7362458
Breast invasive carcinoma, Sarcoma, Lung adenocarcinoma	High	Poor	GO Biological Processes	GO:0071103	DNA conformation change	−44.05	−41.1126749
Breast invasive carcinoma, Sarcoma, Lung adenocarcinoma	High	Poor	GO Biological Processes	GO:0016569	covalent chromatin modification	−36.106	−33.3561787
Breast invasive carcinoma, Sarcoma, Lung adenocarcinoma	High	Poor	Reactome Gene Sets	R−HSA−8953854	Metabolism of RNA	−35.053	−32.3261771
Breast invasive carcinoma, Sarcoma, Lung adenocarcinoma	High	Poor	GO Biological Processes	GO:0051640	organelle localization	−33.013	−30.3084538
Breast invasive carcinoma, Sarcoma, Lung adenocarcinoma	High	Poor	GO Biological Processes	GO:0006403	RNA localization	−32.281	−29.6071533
Breast invasive carcinoma, Sarcoma, Lung adenocarcinoma	High	Poor	GO Biological Processes	GO:0000723	telomere maintenance	−30.21	−27.6000007
Breast invasive carcinoma, Sarcoma, Lung adenocarcinoma	High	Poor	KEGG Pathway	hsa04110	Cell cycle	−28.43	−25.8974939
Breast invasive carcinoma, Sarcoma, Lung adenocarcinoma	High	Poor	GO Biological Processes	GO:0051321	meiotic cell cycle	−28.306	−25.7807397
Breast invasive carcinoma, Sarcoma, Lung adenocarcinoma	High	Poor	GO Biological Processes	GO:0034660	ncRNA metabolic process	−27.081	−24.6019116

Breast invasive carcinoma, Sarcoma, Lung adenocarcinoma	High	Poor	GO Biological Processes	GO:0044839	cell cycle G2/M phase transition	−26.924	−24.451738
Breast invasive carcinoma, Sarcoma, Lung adenocarcinoma	High	Poor	GO Biological Processes	GO:0022613	ribonucleoprotein complex biogenesis	−26.616	−24.1732203
Breast invasive carcinoma, Sarcoma, Lung adenocarcinoma	High	Poor	GO Biological Processes	GO:0051983	regulation of chromosome segregation	−25.963	−23.5544409
Breast invasive carcinoma, Sarcoma, Lung adenocarcinoma	Low	Good	Reactome Gene Sets	R−HSA−156842	Eukaryotic Translation Elongation	−59.22	−55.1373514
Breast invasive carcinoma, Sarcoma, Lung adenocarcinoma	Low	Good	Reactome Gene Sets	R−HSA−163200	Respiratory electron transport, ATP synthesis by chemiosmotic coupling, and heat production by uncou	−25.978	−23.3043942
Breast invasive carcinoma, Sarcoma, Lung adenocarcinoma	Low	Good	CORUM	CORUM:5380	TRBP containing complex (DICER, RPL7A, EIF6, MOV10 and subunits of the 60S ribosomal particle)	−12.331	−9.96735608
Breast invasive carcinoma, Sarcoma, Lung adenocarcinoma	Low	Good	GO Biological Processes	GO:0042254	ribosome biogenesis	−11.259	−8.92368831
Breast invasive carcinoma, Sarcoma, Lung adenocarcinoma	Low	Good	GO Biological Processes	GO:0006839	mitochondrial transport	−10.699	−8.39223942
Breast invasive carcinoma, Sarcoma, Lung adenocarcinoma	Low	Good	GO Biological Processes	GO:0006414	translational elongation	−10.535	−8.23480978
Breast invasive carcinoma, Sarcoma, Lung adenocarcinoma	Low	Good	Reactome Gene Sets	R−HSA−9609507	Protein localization	−10.283	−7.99525236
Breast invasive carcinoma, Sarcoma, Lung adenocarcinoma	Low	Good	GO Biological Processes	GO:0006914	autophagy	−8.811	−6.56167059
Breast invasive carcinoma, Sarcoma, Lung adenocarcinoma	Low	Good	CORUM	CORUM:2920	Respiratory chain complex I (lambda subunit) mitochondrial	−8.7084	−6.46636749
Breast invasive carcinoma, Sarcoma, Lung adenocarcinoma	Low	Good	GO Biological Processes	GO:0042273	ribosomal large subunit biogenesis	−8.3759	−6.14117957
Breast invasive carcinoma, Sarcoma, Lung adenocarcinoma	Low	Good	Reactome Gene Sets	R−HSA−446203	Asparagine N−linked glycosylation	−7.8646	−5.647388
Breast invasive carcinoma, Sarcoma, Lung adenocarcinoma	Low	Good	GO Biological Processes	GO:0051186	cofactor metabolic process	−7.2749	−5.07459484

Breast invasive carcinoma, Sarcoma, Lung adenocarcinoma	Low	Good	GO Biological Processes	GO:1902600	proton transmembrane transport	−6.7205	−4.53007078
Breast invasive carcinoma, Sarcoma, Lung adenocarcinoma	Low	Good	KEGG Pathway	hsa04142	Lysosome	−6.6152	−4.42801991
Breast invasive carcinoma, Sarcoma, Lung adenocarcinoma	Low	Good	GO Biological Processes	GO:0098869	cellular oxidant detoxification	−6.4707	−4.28665988
Breast invasive carcinoma, Sarcoma, Lung adenocarcinoma	Low	Good	GO Biological Processes	GO:0017004	cytochrome complex assembly	−6.3175	−4.13665924
Breast invasive carcinoma, Sarcoma, Lung adenocarcinoma	Low	Good	CORUM	CORUM:2914	Respiratory chain complex I (beta subunit) mitochondrial	−6.1808	−4.00631503
Breast invasive carcinoma, Sarcoma, Lung adenocarcinoma	Low	Good	GO Biological Processes	GO:0006790	sulfur compound metabolic process	−6.1346	−3.96932192
Breast invasive carcinoma, Sarcoma, Lung adenocarcinoma	Low	Good	GO Biological Processes	GO:0097193	intrinsic apoptotic signaling pathway	−6.1211	−3.95890523
Breast invasive carcinoma, Sarcoma, Lung adenocarcinoma	Low	Good	GO Biological Processes	GO:0016197	endosomal transport	−5.8453	−3.70381186
Glioblastoma multiforme, Liver hepatocellular carcinoma	High	Good	GO Biological Processes	GO:0098813	nuclear chromosome segregation	−17.209	−12.8911883
Glioblastoma multiforme, Liver hepatocellular carcinoma	High	Good	GO Biological Processes	GO:0006260	DNA replication	−15.495	−11.8761942
Glioblastoma multiforme, Liver hepatocellular carcinoma	High	Good	GO Biological Processes	GO:0071103	DNA conformation change	−14.338	−10.865929
Glioblastoma multiforme, Liver hepatocellular carcinoma	High	Good	GO Biological Processes	GO:0006281	DNA repair	−14.241	−10.8266664
Glioblastoma multiforme, Liver hepatocellular carcinoma	High	Good	Reactome Gene Sets	R−HSA−1640170	Cell Cycle	−14.19	−10.8264457
Glioblastoma multiforme, Liver hepatocellular carcinoma	High	Good	GO Biological Processes	GO:0010564	regulation of cell cycle process	−11.503	−8.33138457
Glioblastoma multiforme, Liver hepatocellular carcinoma	High	Good	GO Biological Processes	GO:0032392	DNA geometric change	−8.409	−5.41366911
Glioblastoma multiforme, Liver hepatocellular carcinoma	High	Good	GO Biological Processes	GO:0051321	meiotic cell cycle	−8.2257	−5.25062675
Glioblastoma multiforme, Liver hepatocellular carcinoma	High	Good	GO Biological Processes	GO:0051052	regulation of DNA metabolic process	−8.0046	−5.0672514
Glioblastoma multiforme, Liver hepatocellular carcinoma	High	Good	Reactome Gene Sets	R−HSA−156711	Polo−like kinase mediated events	−6.7959	−4.08037827

Glioblastoma multiforme, Liver hepatocellular carcinoma	High	Good	GO Biological Processes	GO:0051383	kinetochore organization	−6.284	−3.62926371
Glioblastoma multiforme, Liver hepatocellular carcinoma	High	Good	GO Biological Processes	GO:0000226	microtubule cytoskeleton organization	−5.8293	−3.24418723
Glioblastoma multiforme, Liver hepatocellular carcinoma	High	Good	GO Biological Processes	GO:0002562	somatic diversification of immune receptors via germline recombination within a single locus	−5.7497	−3.18031693
Glioblastoma multiforme, Liver hepatocellular carcinoma	High	Good	Reactome Gene Sets	R−HSA−2514853	Condensation of Prometaphase Chromosomes	−5.3143	−2.80289726
Glioblastoma multiforme, Liver hepatocellular carcinoma	High	Good	GO Biological Processes	GO:0072331	signal transduction by p53 class mediator	−4.6619	−2.24200639
Glioblastoma multiforme, Liver hepatocellular carcinoma	High	Good	GO Biological Processes	GO:0071897	DNA biosynthetic process	−4.394	−2.02586303
Glioblastoma multiforme, Liver hepatocellular carcinoma	High	Good	GO Biological Processes	GO:0046599	regulation of centriole replication	−4.355	−2.00598845
Glioblastoma multiforme, Liver hepatocellular carcinoma	High	Good	GO Biological Processes	GO:0007062	sister chromatid cohesion	−4.3296	−1.98973799
Glioblastoma multiforme, Liver hepatocellular carcinoma	High	Good	Reactome Gene Sets	R−HSA−68875	Mitotic Prophase	−4.0633	−1.76697608
Glioblastoma multiforme, Liver hepatocellular carcinoma	High	Good	GO Biological Processes	GO:0016570	histone modification	−3.2301	−1.08617462
Glioblastoma multiforme, Liver hepatocellular carcinoma	Low	Poor	KEGG Pathway	hsa05150	Staphylococcus aureus infection	−10.711	−6.393791
Glioblastoma multiforme, Liver hepatocellular carcinoma	Low	Poor	GO Biological Processes	GO:0002274	myeloid leukocyte activation	−9.4058	−5.41801728
Glioblastoma multiforme, Liver hepatocellular carcinoma	Low	Poor	GO Biological Processes	GO:0002253	activation of immune response	−8.8398	−5.12434136
Glioblastoma multiforme, Liver hepatocellular carcinoma	Low	Poor	GO Biological Processes	GO:0032612	interleukin−1 production	−6.8955	−3.85666268
Glioblastoma multiforme, Liver hepatocellular carcinoma	Low	Poor	GO Biological Processes	GO:0006909	phagocytosis	−6.1351	−3.21548936
Glioblastoma multiforme, Liver hepatocellular carcinoma	Low	Poor	Reactome Gene Sets	R−HSA−1280218	Adaptive Immune System	−5.6255	−2.85206888
Glioblastoma multiforme, Liver hepatocellular carcinoma	Low	Poor	GO Biological Processes	GO:0032496	response to lipopolysaccharide	−5.3589	−2.64346436

Glioblastoma multiforme, Liver hepatocellular carcinoma	Low	Poor	GO Biological Processes	GO:0051345	positive regulation of hydrolase activity	−4.6684	−2.07512989
Glioblastoma multiforme, Liver hepatocellular carcinoma	Low	Poor	Canonical Pathways	M4910	ST ERK1 ERK2 MAPK PATHWAY	−4.4279	−1.90656832
Glioblastoma multiforme, Liver hepatocellular carcinoma	Low	Poor	Reactome Gene Sets	R−HSA−449147	Signaling by Interleukins	−4.4248	−1.90656832
Glioblastoma multiforme, Liver hepatocellular carcinoma	Low	Poor	GO Biological Processes	GO:0035690	cellular response to drug	−4.0086	−1.58320078
Glioblastoma multiforme, Liver hepatocellular carcinoma	Low	Poor	GO Biological Processes	GO:0060326	cell chemotaxis	−3.425	−1.14489977
Glioblastoma multiforme, Liver hepatocellular carcinoma	Low	Poor	GO Biological Processes	GO:0019882	antigen processing and presentation	−2.9373	−0.81838156
Glioblastoma multiforme, Liver hepatocellular carcinoma	Low	Poor	GO Biological Processes	GO:0002761	regulation of myeloid leukocyte differentiation	−2.7608	−0.66070143
Glioblastoma multiforme, Liver hepatocellular carcinoma	Low	Poor	GO Biological Processes	GO:0048839	inner ear development	−2.1571	−0.14920018

© 2020 by the authors. Licensee MDPI, Basel, Switzerland. This article is an open access article distributed under the terms and conditions of the Creative Commons Attribution (CC BY) license (http://creativecommons.org/licenses/by/4.0/).
